# Asymmetric transcriptomic signatures between the cob and florets in the maize ear under optimal- and low-nitrogen conditions at silking, and functional characterization of amino acid transporters ZmAAP4 and ZmVAAT3

**DOI:** 10.1093/jxb/erv315

**Published:** 2015-07-01

**Authors:** Xiaoying Pan, Md. Mahmudul Hasan, Yanqiang Li, Chengsong Liao, Hongyan Zheng, Renyi Liu, Xuexian Li

**Affiliations:** ^1^Department of Plant Nutrition, China Agricultural University, Beijing 100193, China; ^2^Shanghai Center for Plant Stress Biology, Shanghai Institutes for Biological Sciences, Chinese Academy of Sciences, Shanghai 201602, China; ^3^University of Chinese Academy of Sciences, Beijing 100039, China

**Keywords:** Cob, florets, maize, nitrogen, transcriptome, ZmAAP4, ZmVAAT3.

## Abstract

Coordinated functioning of the cob and florets of the maize ear confers grain yield. The cob is critical for carbon partitioning and assimilated nitrogen (N) supply for grain development. However, molecular recognition of the cob and peripheral florets, characterization of genes mediating translocation of N assimilates, and responses of these two tissues to low N (LN) remain elusive. Transcriptional profiling of the ear of a maize hybrid at silking in the field revealed 1864 differentially expressed genes between the cob and florets, with 1314 genes up-regulated in the cob and 550 genes up-regulated in florets. The cob was characterized by striking enrichment of genes that are involved in carbon/N transport and metabolism, consistent with the physiological role of the cob in carbon/N storage and transfer during ear development. The florets were characterized by enrichment of hormone signalling components and development related genes. We next examined the response of the cob and florets to LN stress. LN caused differential expression of 588 genes in the cob and only 195 genes in the florets, indicating that the cob dominated the response of the ear to LN at the transcriptional level. LN caused comprehensive alterations such as carbon/N metabolism or partitioning, hormone signalling and protein phosphorylation in terms of gene expression in the cob and/or florets. Fourteen genes responsive specifically to LN provided potential molecular markers for N-efficient maize breeding. We further functionally characterized two newly identified broad-spectrum amino acid transporters, *ZmAAP4* and *ZmVAAT3*, that showed distinct expression patterns in the cob and florets and potentially important roles in amino-N mobilization in the ear. While both proteins could transport various amino acids into yeast or *Arabidopsis* cells, ZmAAP4 appeared to have higher efficiencies than ZmVAAT3 in transporting seven out of 22 examined amino acids.

## Highlight

We performed transcriptomic dissection of the maize cob and its peripheral florets under optimal- and low-nitrogen conditions at silking and functional characterization of amino acid transporters ZmAAP4 and ZmVAAT3.

## Introduction

Maize (*Zea mays* L.) is one of the most important food and feed crops worldwide, and its yield is mostly derived from mature kernels whose production is closely correlated with ear growth at the silking stage (Cantarero *et al.*, 1999). Ear sink capacity, determined by the kernel number and kernel size, is established during a critical period of 2–3 weeks around silking (Schussler and Westgate, 1991; Cantarero *et al.*, 1999). Rather than being merely a supportive tissue of peripheral florets or resulting kernels after pollination, the maize cob serves as a critical sink, providing carbohydrates and nitrogen (N) nutrients for bearing florets and kernels during the reproductive stage (Crawford *et al.*, 1982). Abundant amino acids in the cob are important for carbohydrate portioning in the ear; amino acid interchange and metabolism within the cob at the silking stage plays crucial roles in determining sink strength of the ear and preconditions assimilated-N supply for grain development (Singletary *et al.*, 1990; Below, 1997; Seebauer *et al.*, 2004; Cañas *et al.*, 2009). The soft cob accounts for a significant part of the ear shoot at silking, after which the cob proportion gradually decreases with ear development (Jacobs and Pearson, 1992; Seebauer *et al.*, 2004). Pollination also triggers a local hardening process immediately beneath the pollinated ovule in the cob, and within 5 d after pollination, the outer cob parenchyma gradually develops into hard sclerenchymal tissue (Lenz, 1948). Kernel development and growth depends on the availability of carbon (C) and N assimilates supplied by the cob and the capacity of the kernel to utilize them (Cazetta *et al.*, 1999). Collectively, coordinated functioning of the cob and florets of the maize ear determines grain yield. Insufficient supply of C/N assimilates from the cob may result in kernel abortion and severe yield reduction.

Several genes have been well known for their fundamental functions in mediating ear and/or grain development in maize, such as *Fasciated Ear2*, *Branched Silkless1*, *Glutamine Synthetase* (*Gln1-3* and *Gln1-4*), *Opaque-7*, *Shrunken-2,* and *Empty Pericarp5* (Taguchi-Shiobara *et al.*, 2001; Chuck *et al.*, 2002; Martin *et al.*, 2006; Wang *et al.*, 2011; Hannah *et al.*, 2012; Bommert *et al.*, 2013; Liu *et al.*, 2013). Along with global dissection of different tissues in maize at the metabolic and transcriptomic levels (Kaeppler, 2013; Monaco *et al.*, 2013; Sekhon *et al.*, 2013; Li *et al.*, 2014; Liu *et al.*, 2015), many transcription factors, protein kinases, hormones, and genes associated with starch and storage protein syntheses were shown to be involved in regulation of ear development at the transcriptional level (Liu *et al.*, 2008; Zhu *et al.*, 2009; Wang *et al.*, 2010; Cañas *et al.*, 2011; Amiour *et al.*, 2012; Chen *et al.*, 2014). However, only four cob-specific genes are identified out of 863 organ-specific genes (Sekhon *et al.*, 2011), and molecular characterization of the cob and peripheral florets remains extremely limited. Although it is well known that amino-N interconversion and remobilization in the maize cob is highly dynamic and that amino acid transporters play essential roles in fruit development in plants (Hirner *et al.*, 1998; Okumoto *et al.*, 2002; Rolletschek, *et al.*, 2005; Hammes *et al.*, 2006; Schmidt *et al.*, 2007; Sanders *et al.*, 2009), no genes mediating transport of amino acids within and/or between the cob and florets have been functionally characterized in maize.

At the silking stage, a variety of abiotic stresses negatively affect ear development, resulting in lower fertilization and seed setting rates, reduced assimilate partitioning to the developing ear, and decreased final grain yield (Kiniry and Ritchie, 1985; Schussler and Westgate, 1991; Cantarero *et al.*, 1999; Andrade *et al.*, 2002). N is one of the most important nutrients for maize growth and grain formation, in part by controlling the ability of the kernel to utilize C (Below *et al.*, 2000). N uptake at silking determines kernel number (Andrade *et al.*, 2002; D’Andrea *et al.*, 2008). N deficiency inhibits ear growth and biomass accumulation, and leads to fewer kernels due to reduced cell division, less distal silks, and imbalanced C/N metabolism (Mozafar, 1990; Jacobs and Pearson, 1992; Lemcoff and Loomis, 1994; Seebauer *et al.*, 2004). Previously we showed that low N (LN) affected production of numerous proteins involved in C/N metabolism and hormonal metabolism in young ears at the silking stage (Liao *et al.*, 2012a). However, it is not clear how the cob and florets respond to LN at the transcriptional level. Here, we used the digital gene expression (DGE) analysis to determine gene expression levels in the maize ear grown in the field at the silking stage, and found 1864 differentially expressed genes between the cob and florets. The cob had 1314 up-regulated genes, which were enriched with genes involved in transport facilitation and energy metabolism. We next functionally characterized two broad-spectrum amino acid transporters ZmAAP4 and ZmVAAT3, and found that the former had higher efficiencies in transport of a subset of amino acids. Under LN, the cob dominated the ear response at the transcriptional level, as indicated by 588 and 195 genes differentially expressed in the cob and florets, respectively. Additionally, LN specifically caused differential expression of 14 genes compared to that under phosphorus or potassium deficiency.

## Materials and methods

### Plant materials and growth conditions

Field experiments with a widely grown, high yield maize hybrid DH3719 were conducted following a well established protocol at the Shangzhuang Experimental Station, China Agricultural University, Beijing (Liao *et al.*, 2012a). Sixteen adjacent plots with the same fertility were used for control (optimal N fertilization, ON: 250kg ha^-1^), LN (zero N fertilization), low phosphorus (without application of 135kg P_2_O_5_ ha^-1^), and low potassium (without application of 120kg K_2_O ha^-1^) treatments with four replicates (a total of 16 plots comprising 4 replicates of 4 treatments) (Supplementary Table S1). Control and LN samples were harvested for genome-wide transcriptomic analyses. In addition, low phosphorus and low potassium samples were harvested for identification of genes that were specifically responsive to LN. Soil properties were the same as previously described (Liao *et al.*, 2012a). Three plants with newly emerging silks (~1–5cm) were randomly selected from each plot and harvested early in the morning (6–7 a.m.) at the silking stage, a short period of time when ~50% of plants in the field had silks growing out of the husks. The cob (the central white tissue of the ear) and peripheral florets (the peripheral light yellow tissue of the ear) of the maize ear were harvested separately, flash frozen and stored at −80°C.

### RNA isolation, DGE library construction and sequencing

Total RNA was extracted from the cob and florets of maize plants that were grown under control and LN conditions, respectively, using TRIZOL reagent (Invitrogen). Three biological replicates were used, giving a total of 12 samples (2 tissues × 2 treatments × 3 replicates). From each sample, 6 µg total RNA was used for library construction according to the standard DGE protocol (Eveland *et al.*, 2010). Briefly, mRNA was purified by oligo(dT) magnetic beads adsorption (Invitrogen) and reverse transcribed into cDNAs. The cDNAs were digested with an anchoring restriction enzyme NlaIII, which recognizes and cuts cDNAs at the ‘CATG’ site, and ligated to an Illumina-specific adapter (Adapter 1) at the 5′ end. The junction of Adapter 1 and the CATG site is the recognition site of *Mme*I, which cuts DNA 17bp downstream of this CATG site. Following *Mme*I digestion, cDNAs were purified and a second Illumina adapter, Adapter 2, was ligated to the 3′ ends. After 15 cycles of linear PCR amplification, 95bp fragments containing the cDNA tag and both adapter sequences were purified by 6% TBE PAGE gel electrophoresis and then sequenced by an Illumina HiSeq 2000 sequencer for 49 cycles. Sequencing data (accession no: GSE59613) were deposited in the Gene Expression Omnibus (GEO) database at the National Center for Biotechnology Information.

### Bioinformatics analysis of DGE data

Raw reads (without the ‘CATG’ tag) were first subject to 3′ adapter removal and ‘CATG’ was electronically added to the 5′ end. Only clean reads that contained no ambiguous nucleotide (‘N’), were exactly 21 nt long (CATG + 17bp sequenced cDNA), and had a read count of at least two in a library were retained for further analysis. Clean reads were clustered into unique tags and then mapped to the maize reference genome (v2, www.maizesequence.org) using Bowtie (Langmead *et al.*, 2009), allowing up to one mismatch. Remaining tags were then mapped to the annotated maize transcripts to recover reads that were located at the exon-intron boundaries. Since UTR regions were not well annotated in the maize genome, gene boundaries were extended by 300 nt at both 5′ and 3′ ends to maximize the capture of complete UTRs (Eveland *et al.*, 2010). Tags that were mapped to more than three genes in the genome were removed from consideration. If a tag was mapped to two or three genes, the tag count was divided by the number of mapped genes. The expression value of a gene was calculated as the total count of all tags that were mapped to the sense strand of the gene region.

EdgeR (Robinson *et al.*, 2010) was used to identify the differentially expressed genes between the cob and florets. Differentially expressed genes between LN and control in the cob and florets respectively were also identified to obtain the LN response genes. Data normalization was performed using the trimmed mean of M-values (TMM) method (Robinson and Oshlack, 2010). The method of Benjamini and Hochberg (1995) was used for adjustment for multiple comparisons. A gene was identified as differentially expressed if the false discovery rate (FDR) is <0.05 and its expression level showed at least 2-fold change. A gene was identified as ‘preferentially expressed’ if its normalized expression level was at least 10 TPTM (transcripts per ten million reads) in one sample but was zero TPTM in another sample.

The Gene Ontology (GO) term enrichment of differential expressed genes was conducted using the R package TopGO (Alexa *et al.*, 2006), which included an improved weighted scoring algorithm and Fisher test that was used to determine the significance of enrichment. The functional categorization of differentially expressed genes was visualized using MapMan (Thimm *et al.*, 2004), a Java software used to display genomics datasets onto diagrams of metabolic pathways and other biological processes. The heat map figure was generated with the R package pheatmap (Kolde, 2012) using the normalized gene expression values in each sample.

### Reverse transcription-quantitative real time PCR (RT-qPCR)

RNA purification, cDNA synthesis and RT-qPCR analysis followed previous descriptions (Liao *et al.*, 2012a), with primers listed in Supplementary Table S2. The relative expression level was calculated using the comparative C_T_ method (Livak and Schmittgen 2001) with *ZmUbiquitin* (for maize) and *AtTub4* (for gene overexpression lines) as the internal control. Each treatment had three biological replicates.

### Transient expression of GFP tagged ZmAPP4 and ZmVAAT3

The coding sequences of *ZmAPP4* and *ZmVAAT3* without stop codon were fused to the N-terminus of GFP under the control of the CaMV 35S promoter using the pUC-GFP vector (Takara Bio). The primers for cloning were as follows: ZmAPP4-GFP-F (TCTAGAATGGCGGAGAACAACGTC) and ZmAPP4-GFP-R (GGATCCGGACTTGAACGGG); ZmVAAT3-GFP-F (TCTAGAATGGCGGCGG CGGAGGAG) and ZmAPP4-GFP-R (GGATCCCTAGTAGCTTTCGGCTATCTTG- G). The onion epidermis was placed on the Murashige and Skoog medium [per litre: 4.2g Murashige and Skoog salts (Gibco-BRL), 2% (w/v) Phytablend (Caisson Laboratories), 50mg ampicillin, 30g sucrose, pH 5.7]. Gold particles were coated with 5–10 μg plasmids, washed, resuspended and bombarded into the onion epidermal cells three times (as replicates) for each gene using the Biolistic PDS-1000/He Particle Delivery System (Bio-Rad) according to the manufacturer’s instructions. Transformed cells were incubated at 28°C in the dark for 18h before observation. A confocal microscope (Nikon Eclipse TE2000-E) was used to visualize GPF control and GFP tagged ZmAPP4 and ZmVAAT3. The EZ-C1 (for Nikon C1 Confocal microscope) image software was used for optical sections and Z-stack processing.

### Functional characterization of *ZmAPP4* and *ZmVAAT3* using the 22Δ8AA yeast strain

The *Saccharomyces cerevisiae* mutant 22Δ8AA (gap1-1, put4-1, uga4-1, can1, apl1, lyp1, hip1, dip5), unable to absorb arginine, aspartate, citrulline, GABA, glutamate or proline (Fischer *et al.*, 2002), was used for functional characterization of *ZmAPP4* and *ZmVAAT3*. Coding sequences of *ZmAPP4* and *ZmVAAT3* were cloned into the pDR195 yeast expression vector (with PMA1 promoter and ADH terminator) and transformed into the 22Δ8AA strain, and named as ZmAPP4-pDR-22Δ8AA and ZmVAAT3-pDR- 22Δ8AA, respectively. The primers ZmAPP4-pDR-L (CTCGAGA-TGGCGGAGAACAACGTC) and ZmAPP4-pDR-R (TCAGTAGGACTTGAACGG-G); ZmVAAT3-pDR-L (CTCGAGATGGCGGCGGCGGAGGAG) and ZmVAAT3-pDR-R (GGATCCCTAGTAGCTTTCGGCTATCTTGG) were used for subcloning of *ZmAPP4* and *ZmVAAT3* into the pDR195 vector. The restriction sites used for gene subcloning were XhoI and BamHI. The empty vector pDR195 was transformed into the 22Δ8AA strain (as pDR-22Δ8AA) and the EUROFAN (http://mips.gsf.de/proj/eu- rofan/) wild-type (WT) yeast strain 23344c (MATα, ura3) (as pDR-23344c) as negative and positive controls. The heat shock method was used for yeast transformation (Suga and Hatakeyama, 2005). Transformants were selected on solid agar with yeast N base (YNB) media (6.7g l^-1^) without uracil, supplemented with 0.5g l^-1^ ammonium sulphate and 20g l^-1^ D-glucose. Growth assays were performed in uracil-free YNB media containing 1 or 3mM of Asp, Glu, Pro, Arg, GABA or citrulline as sole N source and 2% D-glucose as C source. The yeast culture with the OD600 value of 1.0 was diluted as 10^–1^, 10^–2^, 10^–3^, 10^–4^ solutions. 10 µl droplets of yeast culture and diluted solutions were spotted onto YNB synthetic media, observed after 3-d incubation at 30°C, and photographed with a digital camera. All experiments were repeated three times with independent colonies of different transformation events.

### Generation and phenotyping of gene overexpression plants

The coding sequence of *ZmAAP4* was amplified with primers ZmAPP4-OE-F (TCTAGAATGGCGGAGAACAACGTC) and ZmAPP4-OE-R (AAGCTTTCAGT-AGGACTTGAACGGG) and that of *ZmVAAT3* was amplified with primers ZmVAAT3-OE-F (TCTAGAATGGCGGCGGCGGAGGAG) and ZmVAAT3-OE-R (AAGCTTCTAGTAGCTTTCGGCTATCTTGG) (Supplementary dataset 1). The PCR products were cloned into pMD19-T vector (Takara Bio), sequenced, digested and ligated into the pSuper1300^+^ vector (with a modified 35S promoter; Yang *et al.*, 2010) using XbaI and HindⅢ sites. The recombinant plasmids pSuper1300^+^-ZmAAP4 and pSuper1300^+^-ZmVAAT3 were then transformed into the *Agrobacterium tumefaciens* strain GV3101. Transformation of *Arabidopsis thaliana* (Col-0) was performed via the floral dip method (Clough and Bent, 1998). Transgenic plants were selected using 35 μg ml^-1^ hygromycin. Resistant T_1_ plants were transferred into the soil (perlite:vermiculite, 1:2), and seeds were harvested from individual plants at maturity for further screen. T_2_ seedlings with ~3:1 segregation ratio (alive:dead) on the selective media were transferred for seed harvest. Transgenic lines were screened until all seedlings were alive on the selective growth media. Three independent homozygous lines (T_3_ in our studies) were selected for further analyses according to the expression level of the transformed gene.

Arabidopsis seeds were surface sterilized, germinated, and grown on ATS agar medium (Schofield *et al.*, 2009) with a day/night period of 16/8h (70 μmol m^-2^ s^-1^) at 23°C±1 after stratifying 48h at 4°C in the dark. After 4 days’ growth, uniform ZmAPP4-OE, ZmVAAT3-OE and Col-0 seedlings were transformed on a square petri dish (13×13cm^2^) containing ATS medium (Schofield *et al.*, 2009) with 1% sucrose, 3mM NO_3_^-^, and one of 22 selected amino acids in appropriate concentrations as the N source. The fresh weight (g), number of leaves per plant, rosette diameter (cm) and primary root length (cm) of *Arabidopsis* seedlings were measured 12 d after transfer. Three petri dishes for each treatment were considered as three independent biological replicates. For each measurement, the average value of four seedlings from a single petri dish was considered as a single replicate. The primary root length was measured using a ruler and expressed in cm. The number of leaves per seedling was counted manually. The rosette diameter of each seedling was measured diagonally twice using a ruler, recording the average value of two measurements on the same rosette in cm. The fresh weight was measured using an electronic balance with 0.1mg resolution (AE 163, Mettler Instrument Corp, Hightstown, NJ).

## Results

A total of >54 million high quality reads were generated by digital gene expression (DGE) to transcriptomically profile the cob and florets of the maize ear at the silking stage under optimal nitrogen (ON) and low nitrogen (LN) conditions. The vast majority of tags (52 340 577) were mapped to protein encoding genes, with others to pseudogenes (1 065 100 tags) and transposable elements (694 843 tags) (Supplementary Table S3). To examine the gene expression with a second approach, we randomly selected 20 genes with various expression levels and differential expression (2-fold change and FDR<0.05) between the cob and florets for RT-qPCR analysis and found the exact same expression patterns for all re-examined genes using these two independent approaches (Supplementary Table S4), indicating that our DGE data were highly reliable.

### Gene expression in the cob and florets at the silking stage

**Fig. 1.**
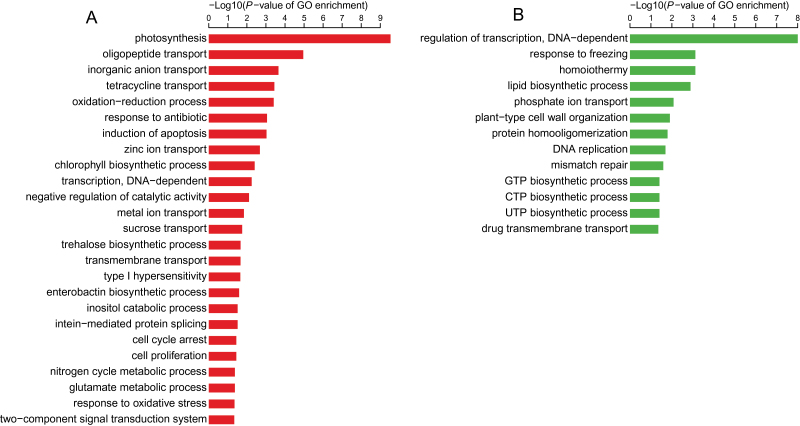
Significantly enriched GO terms in up-regulated genes in (A) the cob versus (B) the florets, under sufficient N supply.

Using FDR<0.05 and 2-fold change as thresholds, we found 1864 genes that were differentially expressed between the cob and florets at the silking stage: 1314 up-regulated in the cob and 550 up-regulated in florets (Table 1; Supplementary Table S5). The differentially expressed genes between the cob and florets were next subject to gene ontology (GO) term enrichment analysis. The up-regulated genes in the cob were enriched with genes involved in transport facilitation, C/N metabolism, and photosynthesis (Fig. 1A). In contrast, most up-regulated genes in florets were involved in cellular processes and lipid biosynthesis (Fig. 1B). Genes related to biotic responses were enriched in both tissues (Fig. 1).

**Table 1.** The number of differentially expressed genes in ears of maize hybrids at silking under different N conditions, revealed by transcriptional profiling

	**Cob vs floret**		**LN vs ON**	
	**ON**	**LN**	**Cob**	**Floret**
Up-regulated genes	1153	920	287	106
Down-regulated genes	442	427	244	63
Differentially expressed genes (≥2-fold change)	1864	1594	588	195

LN, low nitrogen; ON, optimal nitrogen fertilization.

**Fig. 2.**
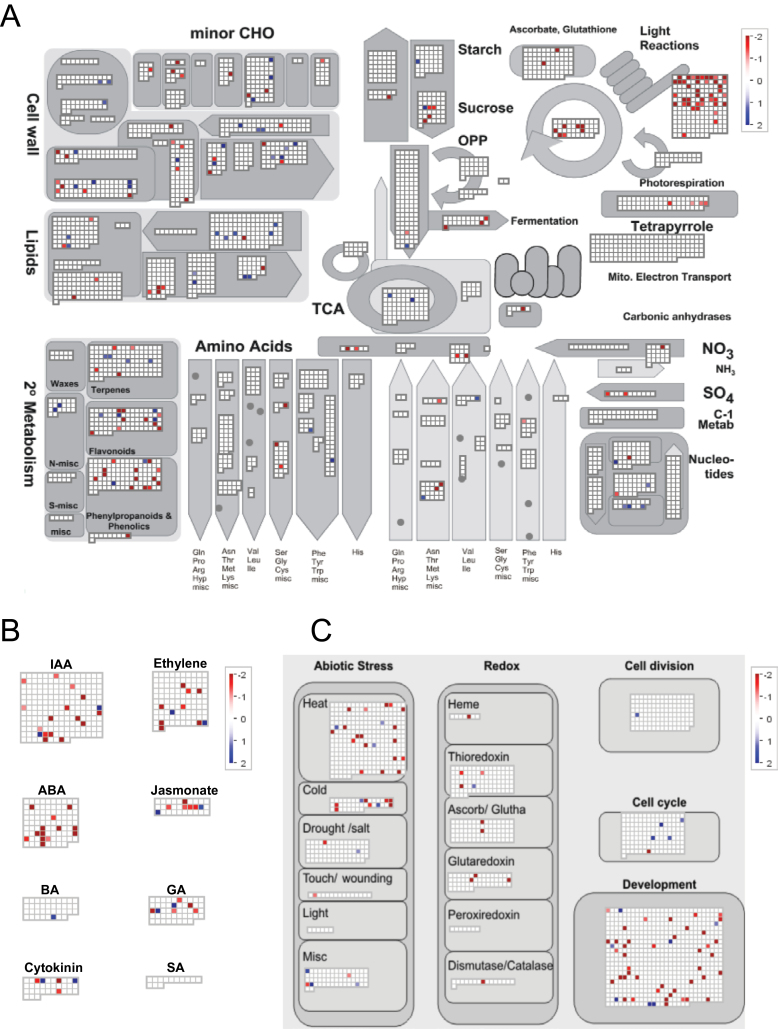
Functional categorization of the differentially expressed genes (FDR<0.05) between the cob and florets under sufficient N supply. Each coloured dot represented one gene. Red colour indicates an increased expression in the cob relative to florets whereas the blue colour indicates an increased expression in florets relative to the cob. (A) Metabolism overview of the differentially expressed genes between the cob and florets. (B) Hormone related genes. (C) Cellular response related genes.

Genes involved in metabolic pathways, hormone and cellular response processes were further analysed using the MapMan programme. Strikingly, genes involved in photosynthesis such as chlorophyll binding (*photosystem II light harvesting complex gene B1B2* and *light-harvesting chlorophyll b-binding protein 3*) and photosystem subunit (*photosystem II subunit P-2*, *photosystem I subunit O*) genes (Fig. 2A; Supplementary Table S5) were unanimously up-regulated in the cob. Amino acid metabolism related gens *GAD2* (*Glutamate decarboxylase 2*, GRMZM2G355906), *a sarcosine oxidas*e (GRMZM2G052266) and *a carboxylyase* (GRMZM2G159149) were up-regulated 8.2-, 5.6-, and 10.2-fold, respectively, in the cob (Fig. 2A; Supplementary Table S5). Genes mediating secondary metabolism such as flavonoid and phenylpropanoid metabolism were also generally up-regulated in the cob (Fig. 2A). Up-regulation of these three categories of genes suggested more active C/N metabolism in the cob tissue than in florets at the silking stage. Notably, several genes regulating ABA synthesis and signalling were up-regulated in the cob, indicating that ABA may be an important regulator for cob development during this stage (Fig. 2B). Different from those up-regulated in the cob, a subset of genes regulating cell wall modification, cell wall cellulose synthesis and cell wall degradation were up-regulated in florets (Fig. 2A). Genes involved in lipid degradation such as *glycerophosphoryl diester phosphodiesterase* (GRMZM2G058- 227) and *fatty acid reductase 5* (GRMZM2G120938) were up-regulated 7.2- and 6.7-fold in florets, respectively (Fig. 2A; Supplementary Table S5). In addition, a gene mediating cell division (GRMZM2G166684) was also up-regulated 2.7-fold in florets. Together with enhanced transcription of *SWITCH1* (GRMZM2G300786) and *cyclin d5;1* (GRMZM2G047637), these results suggested more dynamic cell division, cell wall and membrane formation in florets supporting female flower development in maize (Fig. 2C; Supplementary Table S5).

**Fig. 3.**
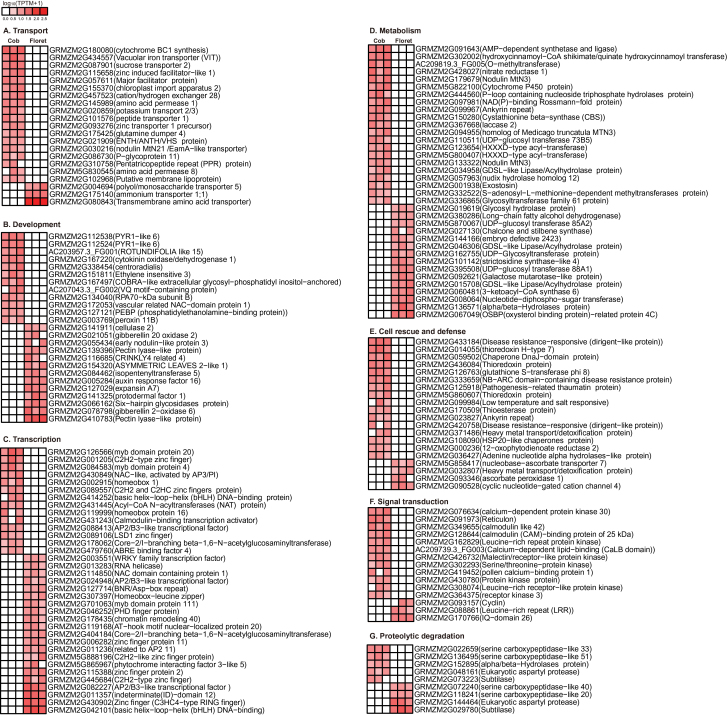
Heat maps showing the expression levels of tissue-specific genes in the cob and florets under sufficient N supply.

Of particular interest were genes only detected in the cob or florets. In total, 63 out of 161 cob-specific genes (Supplementary Table S6) and 45 out of 108 floret-specific genes are functionally unknown genes (Supplementary Table S7), indicating that DGE may identify previously uncharacterized transcripts. We analysed tissue-specific genes with proper annotation using manual functional classification and heat map illustration (Supplementary Tables S6, S7; Fig. 3). First, 18 genes involved in transport facilitation were only detected in the cob, including two carbohydrate transporters, four amino acid transporters, four nutrient transporters and eight other transporters, suggesting the cob’s unique function to transport carbohydrates, amino acids and nutrients for bearing florets (Fig. 3A). Second, 21 genes involved in metabolism also showed cob-specific expression pattern. Third, certain cytokinin and ethylene signalling components, such as *cytokinin dehydrogenase* (*CKX*) and *ethylene-insensitive 3* (*EIN 3*), were only detected in the cob (Fig. 3B). Fourth, 14 transcription factors were only expressed in the cob. In addition, genes involved in cell rescue, signal transduction and proteolytic degradation were also preferentially expressed in the cob during this phase. In sharp contrast, only a few floret-specific genes were related to transport facilitation; three major groups of floret-specific genes were involved in development, transcription and metabolism (Fig. 3). Thirteen development related genes, 21 transcription factors, and 15 metabolism related genes (such as *hydrolyzing O-glycosyl compounds*, *crinkly 4 related 4* (*CR4*), *pectate lyase family protein* and *subtilase family protein*) co-modulated floret development (Fig. 3). *3-ketoacyl-coa synthase 6* and *strictosidine synthase* family proteins were essential for protein synthesis during floret growth. As expected, hormone signalling components *isopentenyl transferase 5*, *gibberellin 2-oxidase 6*, *gibberellin 20 oxidase 2*, and *auxin response factor 16* were specifically expressed in florets (Fig. 3). In summary, the cob and florets showed contrasting gene expression profiles, underpinning their tissue identities and distinct biological functions at a critical developmental stage in maize.

### Effects of low nitrogen supply on global gene expression in the cob and florets

**Fig. 4.**
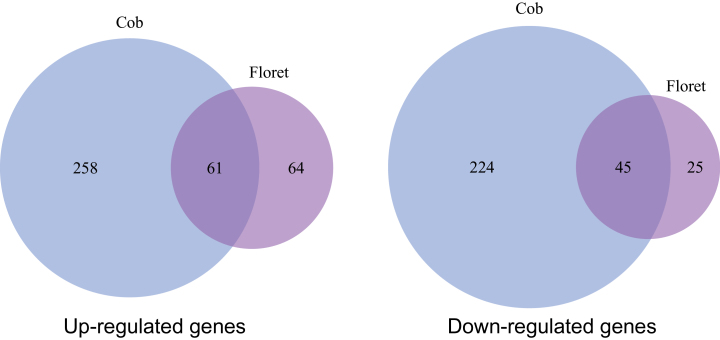
Venn diagram of up-regulated and down-regulated genes in the cob versus the florets, under LN.

LN resulted in differential expression (2-fold cutoff) of 588 genes in the cob and 195 genes in florets at the silking stage (Table 1). For genes with enhanced expression under LN, 258 genes were only detected in the cob, 64 genes only in florets, and 61 genes in both tissues (Fig. 4); for those with reduced expression under LN, 224 genes were detected only in the cob, 25 genes only in florets, and 45 in both tissues (Fig. 4).

**Fig. 5.**
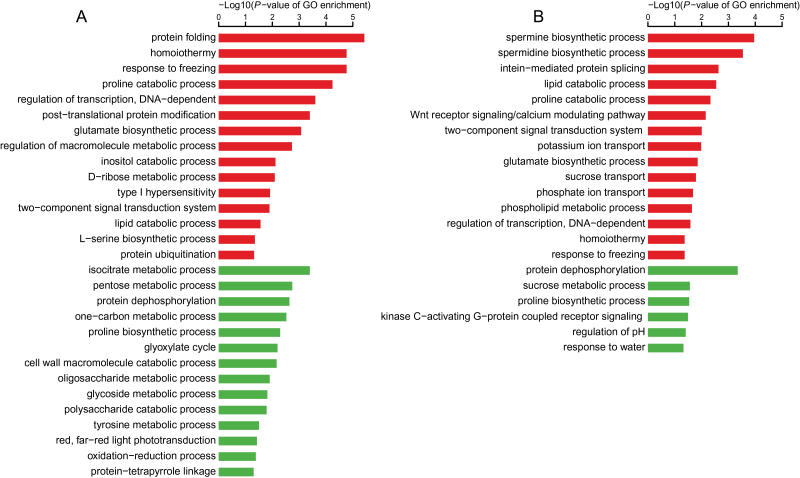
Significantly enriched biological process (BP) GO terms in up-regulated (red) and down-regulated (green) genes in (A) the cob versus (B) the florets, under LN.

GO enrichment analysis showed that among 319 genes up-regulated in the cob in response to LN, genes involved in 15 biological processes were enriched; and among 269 genes down-regulated in the cob, genes involved in 14 biological processes were enriched (Fig. 5A). Among 125 genes up-regulated in the florets in response to LN, genes involved in 15 biological processes were enriched; and among 70 genes down-regulated in the florets, genes involved in six biological processes were enriched (Fig. 5B). The most enriched categories in the cob under LN were carbohydrate and nitrogen metabolic processes (Fig. 5A). Although less significant than those in the cob, genes involved in carbohydrate and nitrogen metabolic processes were still enriched in florets (Fig. 5B), indicating that LN affected these two biological processes in both the cob and florets although different number of genes were affected.

**Fig. 6.**
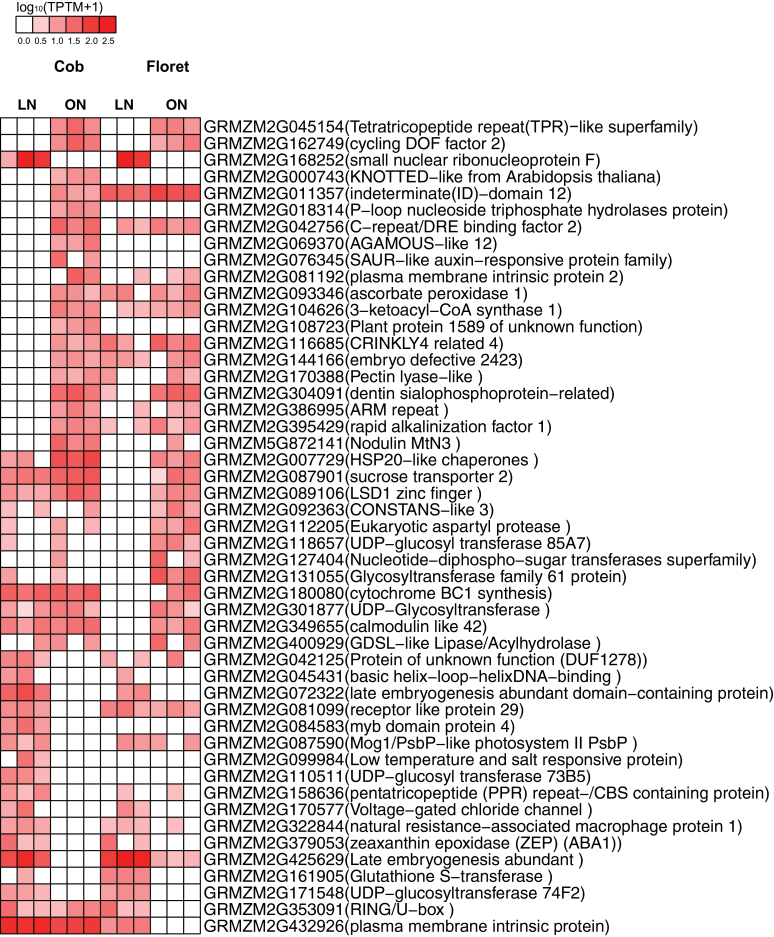
Expression pattern of preferentially expressed genes in the cob or florets under LN.

Further analysing expression of genes mediating C/N metabolism by MapMan visualization showed differential responses of the cob and florets to LN stress (Supplementary Fig. S1; Supplementary Table S8). In the cob, the expression of two genes involved in starch degradation (GRMZM2G34770 and GRMZM2G082034) was repressed. Similarly, *methionine adenosyltransferase* (GRMZM2G117198) was down-regulated 2.3-fold, although *3-phosphoglycerate dehydrogenase* (GRMZM2G073814) was up-regulated 2.5-fold. Genes mediating minor CHO metabolism were differentially regulated in the cob. *Hydrolyzing O-glycosyl compound* (GRMZM2G340656) mediating raffinose synthesis and *trehalose phosphate synthase 11* (GRMZM2G122231) were down-regulated and a *trehalose-6-phosphate phosphatase* (GRMZM2G174396) was up-regulated 3.2-fold. Likewise, secondary metabolism related genes had differential response to LN in the cob. A flavonoid related gene (GRMZM2G099420) was up-regulated 3.5-fold in the cob under the LN condition and the lignin biosynthesis related gene (GRMZM2G443445) was down-regulated 8.4-fold. Notably, many genes regulating photosynthetic light reactions had substantial down-regulation in the cob (Supplementary Fig. S1A), in contrast to unanimous up-regulation of genes in the same process in florets in response to LN (Supplementary Fig. S1B). Transcription of many genes in growth-related processes such as cell wall-modification enzymes *MERISTEM-5* (GRMZM2G392125) and *TOUCH4* (AC210669.3_FG001), and *regulator of chromosome condensation 1* family genes (GRMZM2G337819 and GRMZM2G302245) were also up-regulated in florets under LN. Genes implicated in nucleotide salvage and nucleotide degradation were down-regulated by LN in florets. Most genes involved in minor CHO metabolism, secondary metabolism and amino acid metabolism, except glutamate synthesis and proline degradation, remained unchanged in their expression levels in florets (Supplementary Fig. S1B). Notably, 49 genes had a presence/absence expression pattern in the cob and/or florets under LN (Fig. 6), which were involved in carbohydrate metabolism, nitrogen metabolism, transport facilitation, transcription regulation and cell rescue and defence.

**Fig. 7.**
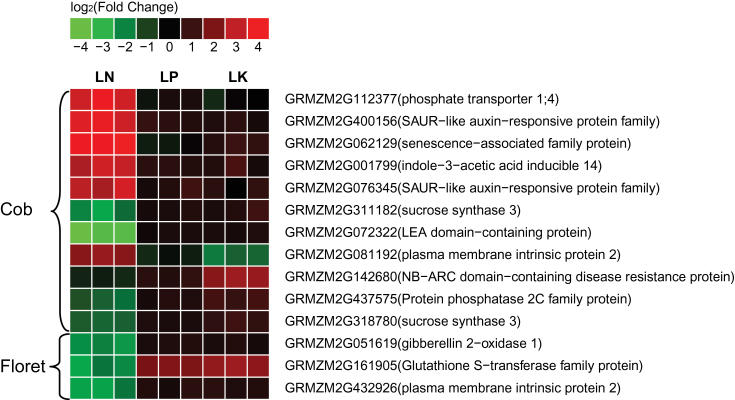
RT-qPCR analysis of selected genes that showed response to LN, but not to low P or low K stress.

Lastly, LN specifically responsive genes (*P*<0.01) were defined as those with significantly differential expression under LN compared to control, while having opposite expression patterns or no differential expression under low phosphorus or low potassium. We comparatively analysed expression levels of a subset of genes closely related to C/N metabolism, hormonal signalling, plant development and defence in the ear using RT-qPCR, and identified six genes with specific up-regulation and eight genes with specific down-regulation under LN in the ear (Fig. 7).

### Functional characterization of two amino acid transporters

**Fig. 8.**
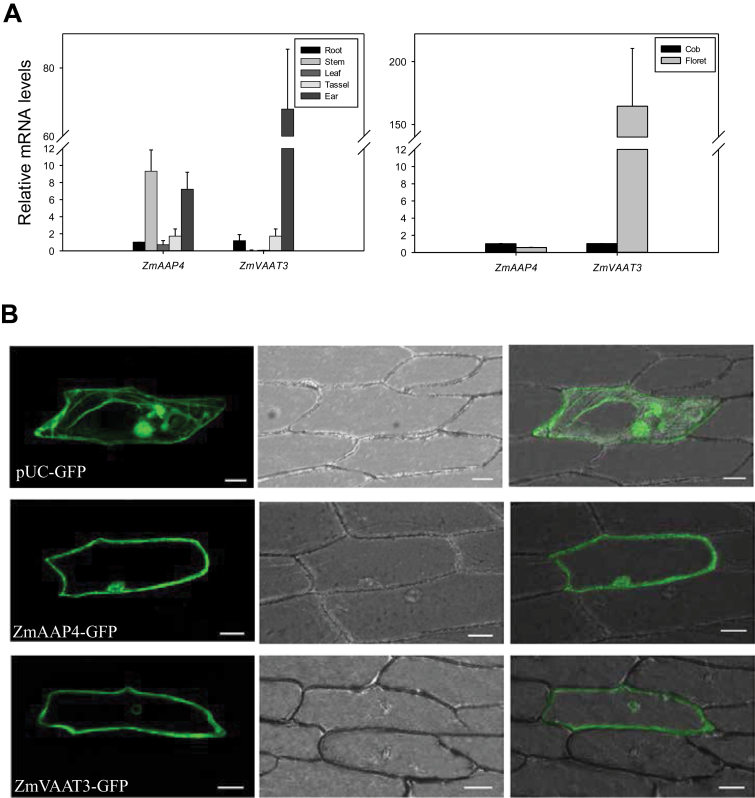
(A) Gene expression levels of *ZmAAP4* and *ZmVAAT3* in different maize tissues. The gene expression level in the root was normalized to 1 when compared with the other four tissues or organs, and that of the cob was normalized to 1 when compared with that of florets within the ear. (B) Subcellular localization of ZmAAP4 and ZmVAAT3 by transient expression of GFP fusion proteins in onion epidermal cells.

Consistent with dynamic assimilated-N translocation and amino acid interconversion in the ear, numerous C/N transport and metabolism genes, including 21 putative amino acid transporters, were found with differential expression in the cob and florets (Supplementary Table S5). To bridge gene expression with physiological functions of amino acid transporters in the ear and comparatively analyse their transport capabilities, we cloned *GRMZM2G110195_T01* and *GRMZM2G080843_T01* for functional characterization. *GRMZM2G110195_T01* (*ZmAAP4*) encodes the putative amino acid permease 4, with 12 transmembrane domains (Supplementary Dataset S1), and had balanced expression in the cob and florets according to the DGE data. *GRMZM2G080843_T01* (*ZmVAAT3*) encodes the putative vacuolar amino acid transporter 3, with 11 transmembrane domains (Supplementary Dataset S1), and was preferentially expressed in florets. At the silking stage, RT-qPCR showed that *ZmAAP4* had higher expression levels in the ear (a similar level in the cob and florets) and stem than in the root, leaf and tassel (Fig. 8A). *ZmVAAT3* was expressed in the root, tassel and ear, but not in the leaf and stem (Fig. 8A). It showed ~70-fold higher expression in the ear compared to that in the root. In the ear, its expression level in the florets is nearly 170-fold higher than in the cob (Fig. 8A). Next, we used GFP tagged constructs to examine where *ZmAAP4* and *ZmVAAT3* are expressed in the cell. We found that when expressed in the onion epidermal cells, ZmAAP4 was mainly located on the plasma membrane and in the nucleus, whereas ZmVAAT3 was mainly located on the plasma and nuclear membranes (Fig. 8B).

**Fig. 9.**
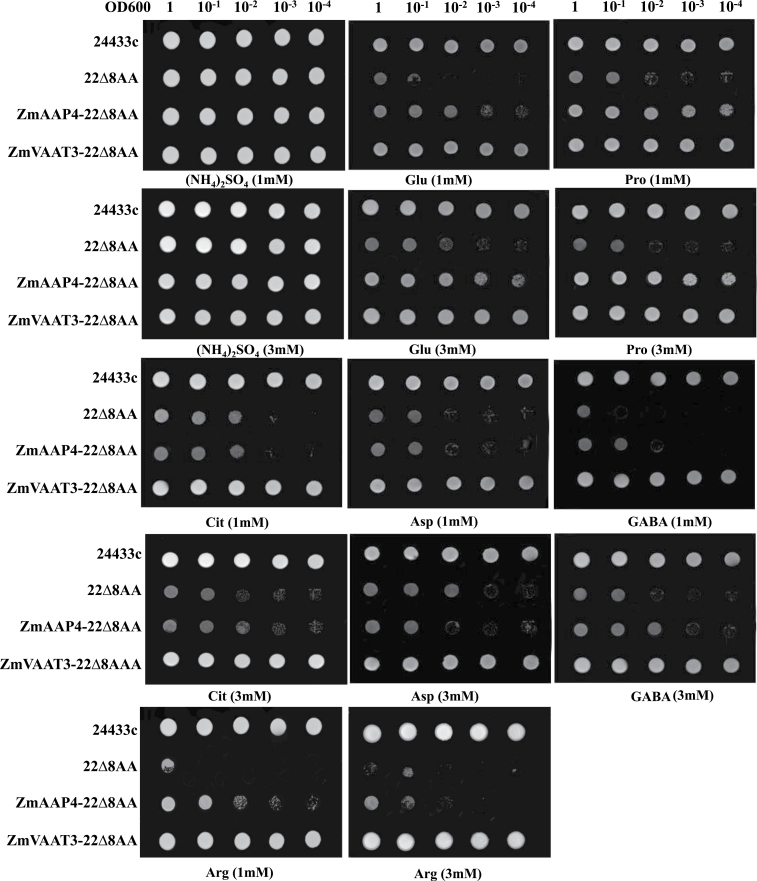
Functional characterization of ZmAAP4 and ZmVAAT3 in the 22∆8AA yeast system. The 23344c and 22Δ8AA served as positive and negative controls, respectively, with the (NH_4_)_2_SO_4_ treatment as a control of N source.

To investigate the transport functionality of ZmAAP4 and ZmVAAT3, we cloned each gene into a pDR195 yeast expression vector and transformed into the yeast mutant strain 22Δ8AA, which is deficient in absorbing arginine, aspartate, citrulline, GABA, glutamate or proline. In sharp contrast to the mutant strain 22Δ8AA itself, the mutant complemented with *ZmAAP4* displayed a similar and robust growth pattern on the media with proline and glutamate and dramatically weaker growth on the media with citrulline, GABA, aspartate or arginine (Fig. 9); the yeast mutant expressing *ZmVAAT3* showed vigorous growth on the six different media specified by the individual amino acid (Fig. 9), indicating that both ZmAAP4 and ZmVAAT3 transported multiple amino acids in the yeast system, although there was a large variation in transport efficiencies.

**Fig. 10.**
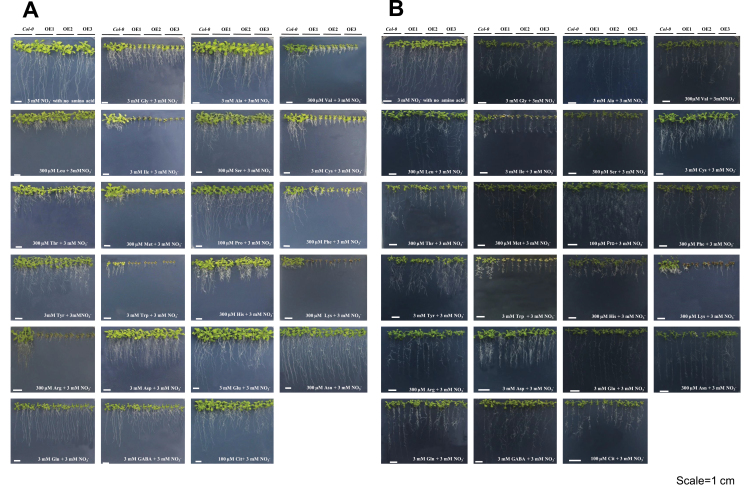
Phenotypes of (A) ZmAAP4-OE and (B) ZmVAAT3-OE *Arabidopsis* seedlings 12 d after transfer onto ATS medium supplemented with different amino acids. The NO_3_^-^ treatment served as a control of N source. OE1, OE2 and OE3 represent three independent gene overexpression lines.

To further characterize their functions in a plant system, we generated transgenic *Arabidopsis* seedlings overexpressing *ZmAAP4* (ZmAAP4-OE) or *ZmVAAT3* (ZmVAAT3-OE), which were grown on a series of ATS media (Schofield *et al.*, 2009) containing different basic amino acids for 12 d for morphological examination. These seedlings showed dramatic alterations in primary root length in many media supplemented by individual amino acids (Fig. 10; Supplementary Tables S10, S11). Compared to WT plants, ZmAAP4-OE seedlings showed reduced primary root growth on media with 17 amino acids except Ala, Ser, Tyr, Asp and Asn (Fig. 10A; Supplementary Tables S9, S11); however, ZmVAAT3-OE seedlings had enhanced primary root length on media supplemented with Leu, Ala, Val, Phe, Met, Arg, Asn or Glu and reduced growth on media with Ser, Cys, Thr, Trp, Lys, Gln, Cit, GABA or His (Fig. 10B; Supplementary Tables S10, S11). Either stimulatory or inhibitory effects of amino-N on primary root growth in ZmAAP4-OE and ZmVAAT3-OE lines suggested substantial uptake of corresponding amino acids mediated by ZmAAP4 and ZmVAAT3 *in vivo*. In addition to alterations in primary root growth, the number of rosette leaves in ZmAAP4-OE and ZmVAAT3-OE seedlings also varied compared to WT seedlings: ZmAAP4-OE seedlings had more rosette leaves on media with Met, Tyr, Asp, Glu or Asn and less leaves on media containing 17 out of 22 tested amino acids except Met, Tyr, Asp, Glu and Asn (Supplementary Tables S9, S11); ZmVAAT3-OE seedlings had more rosette leaves on media containing Leu, Phe, Tyr or Gln, and less leaves on media with 16 amino acids except Leu, Phe, Tyr, Asp, Gln and GABA (Supplementary Tables S10, S11). The rosette diameter of ZmAAP4-OE seedlings significantly increased on the medium with Ala and decreased on media with 14 amino acids except Ala, Leu, Thr, His, Asp, Asn, Gln and Cit (Supplementary Tables S9, S11); the rosette diameter of ZmVAAT3-OE seedlings significantly increased on media supplemented with Ala and decreased on media containing Gly, Val, Ile, Ser, Tyr, Trp, His, Lys, Glu, Gln or Cit (Supplementary Tables S10, S11). Not surprisingly, the fresh weight of ZmAAP4-OE seedlings increased on the medium supplemented with Ala or Leu and decreased on media with 17 amino acids except Ala, Leu, Asp, Asn or Gln (Supplementary Tables S9, S11); while the fresh weight of ZmVAAT3-OE seedlings significantly increased on media with Met, Pro, Phe or Asn and decreased on media containing Ile, Ser, Cys, Trp, His, Lys, Arg, Glu, Gln or GABA (Supplementary Tables S10, S11).

## Discussion

### Asymmetric transcriptomic signatures of the cob and florets in the maize ear

To date, there has been no in-depth characterization of the cob and peripheral florets at the molecular level. Predominant enriched GO terms were related to transport facilitation in the cob, underpinning the nature of the cob as a source tissue providing C and N for floret development and growth during the silking stage (Fig. 1). Sucrose is a main carbohydrate intermediate in maize (Hofstra and Nelson, 1969). Different from strong expression of sucrose transporter 2 in sink leaves in tomato (Barker *et al.*, 2000), specific expression of sucrose transporter 2 (GRMZM2G087901) may promote carbon transport from the cob to florets at the silking stage (Fig. 3). Peptide transport is a more efficient means of N allocation than amino acid transport (Higgins and Payne, 1982). The peptides can be rapidly hydrolysed by peptidases as a source of amino acids, N or C in plants (Koh *et al.*, 2002). *PTR1* is a crucial high affinity peptide transporter in *Arabidopsis* (Komarova *et al.*, 2008). *ZmPTR1* mediates small peptide transport from the endosperm into the embryo in maize, and its overexpression promotes plant growth when grown on a medium with Ala-Ala dipeptide as the unique N source (Tnani *et al.*, 2013). Specific expression of *PTR1* in the cob at the silking stage indicated dynamic amino acid and peptide translocation towards florets (Fig. 3). Serine carboxypeptidase-like proteins play a role in mobilization of seed storage reserves in barley (Degan *et al.*, 1994). Serine-type carboxypeptidases (GRMZM2G057611 and GRMZM2G101576) may promote nutrient mobilization from the cob to florets in maize (Fig. 3). Additionally, potassium and zinc transport from the cob to florets, mediated by potassium transporter 2/3, zinc induced facilitator-like 2 and zinc transporter 12 precursor, were also indispensible for floret development (Fig. 3; Kim *et al.*, 1998; Haydon and Cobbett, 2007). The most enriched biological process GO term was photosynthesis in the cob, with a significant *P*-value of 9.60e-12 (Fig. 1). Our TopGO analysis showed that out of 1314 up-regulated genes in the cob, 21 were involved in photosynthesis. Photosynthesis genes may be involved in energy transfer or metabolism, supporting highly dynamic C and N transport activities within the cob (‘source’) or towards florets (‘sink’) (Thomas and Rodriguez, 1994; Rajcan and Tollenaar, 1999). Alternatively, these genes may have previously uncharacterized functions in the cob, as many genes have multiple functions beyond original annotation. Indeed, a large number of photosynthesis genes were expressed in the root in *Arabidopsis* and rice (Himanen *et al.*, 2004; Lian *et al.*, 2006) although their biological functions remain to be elucidated.

Two major types of genes preferentially expressed in florets were hormone signalling components and developmental regulators, which collectively regulated floret development and growth (Fig. 3). Cytokinin plays essential roles in determining sink strength and grain production in crop plants (Ashikari *et al.*, 2005). *IPT* was specifically expressed in florets, which regulated floret development and kernel formation via controlling cytokinin biosynthesis (Medford *et al.*, 1989; Gan and Amasino, 1995; Brugiere *et al.*, 2008). Specific expression of *gibberellin 2-oxidase 6* and *gibberellin 20 oxidase 2*, *auxin response factor 16*, coordinated to promote floret growth (Dellaporta and Calderon-Urrea, 1994; Sasaki *et al.*, 2002; Wang *et al.*, 2005). Interestingly, a series of genes related to reproductive development were specifically expressed in florets. The maize *CR4* gene encodes a serine/threonine receptor-like kinase that controls a series of developmental processes in seed formation and embryo morphogenesis (Becraft *et al.*, 1996). *ASL* plays a role in cell fate determination in flowers (Li *et al.*, 2005). Genes encoding plastocyanin-like domain-containing protein and subtilase family protein mediate seed development (Haldrup *et al.*, 1999; D’erfurth *et al.*, 2012). *AP2/ERF* family genes act as transcription activators in promoting cell proliferation, differentiation, and morphogenesis, especially during embryogenesis in *Arabidopsis* (Yamaguchi-Shinozaki and Shinozaki, 2006; Iwase *et al.*, 2011). Floret specific expression of *RAP2.11* might suggest its role in mediating floret differentiation in maize. Twenty-one transcription factors, especially bZIP and MYB family proteins, might respond to hormone signalling and play a significant role in regulating grain filling (Fig. 3; Jakoby *et al.*, 2002; Dubos *et al.*, 2010).

Taken together, our results suggested that tissue-specific gene expression played fundamental roles in mediating tissue differentiation and development. Consistent with their developmental functions at the silking stage, the cob was characteristic of preferential expression of genes related to C/N transport and metabolism, serving as a major C/N source for floret development; while florets favoured preferential expression of hormone signalling components and development related genes, supporting floret post-initiation development. Preferential expression of certain C/N metabolism related genes in florets indicated that these genes were necessary for floret build-up following initial differentiation. Our finding also substantially expanded the current spectrum of how to define the cob and florets at the transcriptional level, providing valuable tissue-specific marker genes for further functional characterization.

### The cob had a dominant role at the transcriptomic level in the ear at the silking stage

The cob plays fundamental roles during ear development by providing C and N nutrients or assimilates for floret development and grain filling, and sink strength of the ear is closely associated with amino acid interchange and metabolism in the cob at the silking stage (Cliquet *et al.*, 1990; Ma and Dwyer, 1998; Seebauer *et al.*, 2004; Cañas *et al.*, 2009). Despite the functional importance of the maize cob, only four cob-specific genes were identified in maize transcriptomic profiling, in sharp contrast to the other 859 organ-specific genes (Sekhon *et al.*, 2011). We found 1314 genes with significant (≥2-fold change) up-regulation in the cob with sufficient nutrient supply, which was ~2.3-fold as many as those up-regulated in florets (Table 1), together with more tissue specifically expressed genes in the cob (Supplementary Tables S6, S7), suggesting obvious dominance of the cob in the ear in terms of gene activation at the silking stage. Dominance of the cob in gene activation at the silking stage was in agreement with its function as a crucial source tissue supporting floret development during a critical developmental stage. Further, 3-fold genes (588 genes) had differential expression in the cob under LN, compared with 195 differentially expressed genes in florets (Table 1; Fig. 4), indicating that the cob dominated the ear response to N limitation at the transcriptomic level at the same critical developmental stage. Even for GO enrichment analysis, enriched biological processes in the cob were significantly more than those in florets (Fig. 5), corroborating that the cob responded to N limitation in a dominant manner, with florets as a secondary player in terms of gene regulation. One possible reason was that the cob, as the crucial tissue supporting differentiation and development of florets, was much more sensitive to LN than the florets. Given the dominant role of the cob in the response to LN in the ear at silking, we expected more genes involved in metabolic processes identified in the cob with differential expression under the LN condition. Indeed, we found many genes involved in starch cleavage, minor CHO metabolism and secondary metabolic processes in the cob under LN, with few identified in florets under LN during this stage (Supplementary Fig. S1). Also, less differentially expressed genes regulating amino acid metabolism and biotic stress processes were detected in florets than in the cob under the LN condition. In plants, the cell wall plays an important role in various physiological processes such as growth, intercellular communication and interaction with the environment (Cosgrove, 2005). Genes regulating macromolecule catabolic and anabolic processes in the cell wall were differentially regulated in the cob rather than in florets under LN. It is well known that LN significantly reduces ear growth, biomass accumulation in the ear after silking and the kernel number by reducing sink strength and altering C metabolism (Uhart and Andrade, 1995a, *b*; Andrade *et al.*, 2002; Paponov *et al.*, 2005; Méchin *et al.*, 2007; Liao et al., 2012a, b). Misregulation of numerous genes mediating C/N metabolism and transport in the cob (Fig. 5, Supplementary Fig. S1; Supplementary Table S8) perfectly interpreted negative physiological effects of LN on ear growth and kernel development. Likewise, more genes involved in the cell rescue and response such as far red light photo-transduction and oxidation reduction processes were detected in the cob (Fig. 5; Supplementary Table S8). Consistent with these observations, ribosomal protein family genes were differentially regulated in the cob under LN (Supplementary Table S8). Further, expression alterations of genes involved in transcription facilitation and signal transduction in the cob represented a tissue-preferential primary response of the regulatory machineries to LN. Taken together, our results suggest that the cob is the dominant tissue in the ear in terms of gene transcription at the silking stage, and it at least numerically dominates the ear response to LN as well.

### Low nitrogen had comprehensive impacts on gene expression in the ear

LN severely inhibits maize growth and significantly reduces grain yield. Under LN, genes involved in the glutamate biosynthetic process, proline catabolic process, lipid catabolic process, homoiothermy, response to freezing, regulation of DNA dependent transcription and two component signal transduction were up-regulated (Fig. 5); and genes mediating protein dephosphorylation and proline biosynthesis were down-regulated in both tissues (Fig. 5), indicating complicated and concerted responses of the cob and florets to the LN stress. N deficiency affects N and sugar metabolism and/or partitioning between source and sink tissues (Hermans *et al.*, 2006). Glutamine (Gln), glutamate (Glu), and 2-oxoglutarate are major components of the N-sensor system to monitor accumulation of different ‘checkpoint’ molecules of NO_3_^-^ and NH_4_^+^ in higher plants (Kang and Turano, 2003). Proline biosynthesis is regulated by the N flux and functions in osmo-regulation and other stress responses by controlling the level of P5CS (Kishor *et al.* 1995; Székely *et al.*, 2008). Up-regulation of glutamate biosynthesis, proline catabolic process and down-regulation of proline biosynthesis may indicate internal N status, similar to the indicative role of Asn/Gln in the cob (Seebauer *et al.*, 2004), or serve as a potential mechanism to alleviate N deficiency by accelerating amino acid metabolism and internal N exchange under LN in the ear during the silking stage. In particular, Glu is among those amino acids critical for carbohydrate partitioning in the ear at the silking stage, and its metabolism in the cob partially preconditions assimilated-N supply for grain development and growth (Seebauer *et al.*, 2004; Cañas *et al.*, 2009). LN may negatively affect C/N allocation towards the developing florets by altering glutamate and proline metabolism in this study. Glutamate and proline metabolism might also have a similar role in mediating kernel set and filling as *Gln1-3* and *Gln1-4* do (Martin *et al.*, 2006); thus, LN-directed abnormal glutamate and proline metabolism partially contributed to reduction in the kernel size and number. Alternatively, amino acids may form conjugates with IAA to regulate ear growth and development under the low N condition as they function to stimulate cell or tissue elongation (Feung *et al.*, 1977; Staswick *et al.*, 2005). The lipid catabolic process was also up-regulated in both tissues under LN (Fig. 5), similar to previous report in *Arabidopsis* (Martin *et al.*, 2002). Protein phosphorylation plays an important regulatory role in signal transduction and control of enzyme activities under changing N conditions (Engelsberger and Schulze, 2012). Enrichment of ‘protein phosphorylation’ terms suggested that protein phosphorylation may activate certain N assimilatory pathways for ear development under the LN condition, and dephosphorylation may inactivate downstream targets (Fig. 5). The NR (nitrate reductase) activity is post-translationally regulated by a phosphorylation–dephosphorylation mechanism (Kaiser and Huber, 1994; Bachmann *et al.*, 1996). *CHL1*, a dual affinity nitrate transporter and a nitrate sensor, also functions depending upon phosphorylation by the protein kinase CIPK23 under low nitrate conditions (Ho *et al.*, 2009). In this study, the *phosphatase 2C* (GRMZM2G159811) was significantly repressed in the cob and florets; the *calcium-dependent phosphotriesterase* (AC233955.1_FG008) was upregulated 10.7-fold in florets and a *phosphatase-related* gene (GRMZM2G149704) was upregulated 2.9-fold in the cob (Supplementary Table S8). Therefore, protein dephosphorylation may be very important to activate N assimilation supporting ear development in maize under LN.

Importantly, we identified 14 genes specifically responsive to LN compared to the low phosphorus or potassium treatment (Fig. 7), indicating direct effects of LN on N metabolism and allocation. Glutathione S-transferases (GSTs) function in regulation of cellular metabolism and are involved in a variety of stress responses (Marrs, 1996). Specific down-regulation of the *glutathione s-transferase* in florets probably indicated reduced nitrogen metabolism as an adaptive response to insufficient N supplies (Fig. 7). Closely related to N nutritional status is carbohydrate partitioning that modulates plant growth and development over the entire life cycle (Chiou and Bush, 1998; Hermans *et al.*, 2006). LN generally promotes carbon allocation towards the root to stimulate lateral root emergence and overall root growth in contrast to suppressive effects of K deficiency on root growth (Hermans *et al.*, 2006). *The sucrose synthase 3* plays a major role in sucrose entry into diverse pathways involved in cellular processes (Fu and Park, 1995). Up-regulation of two *sucrose synthase 3* genes in the cob in response to N limitation suggested enhanced sucrose synthesis and metabolism probably towards florets. A previous systemic screen of N nutritional biomarkers revealed 112 genes in maize, with no hormone biosynthesis related genes (Yang *et al.*, 2011). However, we found that auxin and GA signalling components were also specifically responsive to LN, indicating that auxin and GA may be closely linked to N signalling in the maize ear. Potentially, coordinated modulation of auxin and GA signalling may reprogramme cell division or expansion in the maize ear under LN at the silking stage. Notably, *phosphate transporter 1;4* may be an biomarker indicating N status in maize (Yang *et al.*, 2011). N deficiency also induced up-regulation of the senescence-associated gene, similar to previous report in *Arabidopsis* (Peng *et al.*, 2007), suggesting that the senescence-associated gene may have novel functions in plant response to N deficiency or somehow promote ear premature development under LN. Other non-nutrient stress related genes either have multiple functions in diverse environmental stresses, or indicate that the LN stress triggers a N specific response, together with a general stress response applied to a wide array of other biotic and abiotic stresses (Fujita *et al.*, 2006; Atkinson and Urwin, 2012; Liao *et al.*, 2012a, b). Identification of 14 LN specifically responsive genes provided putative molecular markers for N deficiency diagnosis in the field and N efficient maize breeding in the long run.

### ZmAAP4 and ZmVAAT3 were broad-spectrum amino acid transporters with differential substrate selectivity and transport

Dynamic amino acid metabolism is a crucial feature of the quickly developing maize ear at the silking stage (Mozafar, 1990; Jacobs and Pearson, 1992; Lemcoff and Loomis, 1994; Seebauer *et al.*, 2004). Amino acid transporters may be responsible for amino-N transfer from the vegetative tissues towards the developing ear; or more importantly, they operate downstream of amino acid metabolism to mediate N allocation and carbon partitioning during ear development and growth as a fundamental biological process (Seebauer *et al.*, 2004; Cañas *et al.*, 2009, 2011). However, no amino acid transporters have been functionally characterized in maize. We demonstrated that ZmAAP4 and ZmVAAT3 were broad-spectrum amino acid transporters with different transport efficiencies, as indicated by large phenotypic variations among gene overexpression lines (Fig. 10). This conclusion was consistent with the notion that AAP and VAAT members are broad-spectrum amino acid transporters (Fischer *et al.*, 1995; Su *et al.*, 2004). Among 22 amino acids transported by ZmAAP4 and ZmVAAT3, Asp, Asn, Glu, Gln, Arg, Ala, Val, Ser and His are the most abundant amino acids in the cob; Asp, Asn, Glu, Gln, Arg, Ala, Ser and Pro are critical for carbohydrate partitioning in the ear at the silking stage (Seebauer *et al.*, 2004). Enzymatic interconversions among Gln, Ala, Asp and Asn in the cob precondition assimilated-N supply for grain development and growth (Seebauer *et al.*, 2004; Cañas *et al.*, 2009). Essential physiological or biological functions of amino acids transported by ZmAAP4 and ZmVAAT3 in the ear suggested that ZmAAP4 and ZmVAAT3 played important roles in promoting amino-N mobilization in and between the cob and florets during tissue differentiation and embryo and organ genesis in the ear. Floret-preferential expression of *ZmVAAT3* (Fig. 8A) further indicated its potential functions in mediating amino acid fluxes, storage or other metabolic processes during kernel development. Notably, both ZmAAP4 and ZmVAAT3 were able to transport nine amino acids essential for human development, including Lys, Trp, Met, Phe, Val, Thr, Leu, Ile and His (Fürst, 1998). Most cereal grains contain very limited amount of Lys, Trp and Met (Galili *et al.*, 2002). High-Lys LY038 maize is grown in a number of countries and quality protein maize, enriched in Lys and Trp, is under development through genetic engineering (Glenn, 2007). Transport of Lys, Trp and Met by ZmAAP4 and ZmVAAT3 is potentially important for enhancing nutritional values of maize grains as a staple food and feed resource. *ZmAAP4* and *ZmVAAT3* may therefore serve as valuable target genes for maize breeding given large genetic variations in gene expression and regulation in maize germplasms across the world.

Importantly, ZmAAP4 may have higher efficiencies than ZmVAAT3 in transporting seven amino acids, including five nonpolar amino acids (Phe, Pro, Met, Leu and Val), basic amino acid Arg (pH 6–7), and acidic amino acid Glu (pH 6–7), given similar gene expression levels in different transgenic lines (Supplementary Fig. S2). Negative effects of Phe, Pro or Met on fresh weights of ZmAAP4-OE seedlings were in sharp contrast to positive effects of these three amino acids on the same measurement of ZmVAAT3-OE seedlings (Supplementary Tables S9, S10), implying over-uptake of Phe, Pro or Met due to a higher transport efficiency of ZmAAP4 than that of ZmVAAT3, given the consistent results from three representative biological replicates. Phe, Met, Leu, Val, Arg or Glu stimulated longitudinal growth of the primary root of ZmVAAT3-OE seedlings; by contrast, they reduced primary root length of ZmAAP4-OE seedlings probably due to amino-N over-uptake and consequent disruption of C/N metabolic homeostasis, corroborating the higher transport efficiency of ZmAAP4 (Supplementary Tables S9, S10). Consistently, Phe or Leu reduced the rosette leaf number in ZmAAP4-OE seedlings, in contrast to stimulation of rosette leaf proliferation in ZmVAAT3-OE seedlings (Supplementary Tables S9, S10). One possible interpretation could be that relatively preferential expression of *ZmVAAT3* in the ear (Fig. 8A) made it more substrate selective, resulting in low efficiencies in mediating transport of a subset of amino acids. On the other hand, *ZmAAP4* had more comparable expression in all five tissues (Fig. 8A), indicating less substrate selectivity and higher transport efficiencies. This observation was also consistent with a previous report that AAP members were more efficient than VAATs (Su *et al.*, 2004).

Although, as previously discussed, functional investigation using gene overexpression lines showed larger transport capacities of ZmAAP4 under certain circumstances, ZmVAAT3 was a stronger transporter than ZmAAP4 in terms of Cit, GABA, Asp and Arg uptake according to yeast complementation data (Fig. 9). More robust growth of the *ZmVAAT3* complemented 22Δ8AA strain than that of the *ZmAAP4* complemented strain on media with Cit, GABA, Asp or Arg also implied ZmVAAT3’s higher affinity to these very different amino acids in a yeast system. Indeed, ZmAAP4 and ZmVAAT3 had quite different substrate selectivity as well as transport efficiencies as shown by their differential phenotypes (Fig. 10). Down-regulation of fresh weights of ZmVAAT3-OE seedlings (by Gln) and ZmAAP4-OE seedlings (by Gly, Val, Ile, Ser, Cys, Thr, Met, Pro, Phe, Tyr, His or Cit) were likely derived from variations in substrate affinity or transport efficiencies of these two transporters confronting different amino-N cargoes (Fig. 10; Supplementary Tables S9, S10). Similarly, Ser inhibited primary root growth of ZmVAAT3-OE seedlings, while Gly, Ile, or Pro inhibited that of ZmAAP4-OE seedlings (Fig. 10; Supplementary Tables S9, S10), indicating substrate differentiation and efficiency variation of two transporters. This argument was also well supported by contrasting effects of many other tested amino acids on the leaf number and rosette diameter of ZmVAAT3-OE and ZmAAP4-OE seedlings (Fig. 10; Supplementary Tables S9, S10).

## Supplementary data

Supplementary data are available at *JXB* online.

Supplementary Figure S1. Metabolism overview of the differentially expressed genes in the cob and florets under LN.

Supplementary Figure S2. Transcription levels of *ZmAAP4* and *ZmVAAT3* in gene overexpression lines.

Supplementary Table S1. Fertilization practice among different nutrient treatments.

Supplementary Table S2. Primer sequences used for RT-qPCR.

Supplementary Table S3. Total number of tags and mapped genes from the DGE sequencing data.

Supplementary Table S4. Validation of the expression fold change of selected genes from DGE data by RT-qPCR.

Supplementary Table S5. List of differentially expressed genes between the cob and florets under sufficient nitrogen supply.

Supplementary Table S6. List of genes that were preferentially expressed in the cob with sufficient nitrogen supply.

Supplementary Table S7. List of genes that were preferentially expressed in florets with sufficient nitrogen supply.

Supplementary Table S8. List of differentially expressed genes in the cob and florets under LN.

Supplementary Table S9. Quantification of phenotypes of ZmAAP4-OE *Arabidopsis* seedlings 12 d after transfer onto ATS medium supplemented with 3mM NO_3_^-^ and different amino acids.

Supplementary Table S10. Quantification of phenotypes of ZmVAAT3-OE *Arabidopsis* seedlings 12 d after transfer onto ATS medium supplemented with 3mM NO_3_^-^ and different amino acids.

Supplementary Table S11. Summary of effects of 22 amino acids on growth parameters of ZmAAP4-OE and ZmVAAT3-OE *Arabidopsis* seedlings.

Supplementary Dataset S1. Coding sequences and functional domains of *ZmAAP4* and *ZmVAAT3*.

Supplementary Data

## Abbreviations:

C: carbon
DGE: digital gene expression
FDR: false discovery rate
GO: gene ontology
LK: low potassium
LN: low nitrogen
LP: low phosphorus
ON: optimal nitrogen
RT-qPCR: reverse transcription-quantitative PCR.
