# Supplementary material for: Asymmetric transcriptomic signatures between the cob and florets in the maize ear under optimal- and low-nitrogen conditions at silking, and functional characterization of amino acid transporters ZmAAP4 and ZmVAAT3
Source: J Exp Bot. 2015 Jul 1;66(20):6149–66. doi: 10.1093/jxb/erv315 (PMC4588875; doi:10.1093/jxb/erv315)
Supplement: Supplementary Data [file supp_66_20_6149__index.html]

Asymmetric transcriptomic signatures between the cob and florets in the maize ear under optimal- and low-nitrogen conditions at silking, and functional characterization of amino acid transporters ZmAAP4 and ZmVAAT3 — Asymmetric transcriptomic signatures between the cob and florets in the maize ear under optimal- and low-nitrogen conditions at silking, and functional characterization of amino acid transporters ZmAAP4 and ZmVAAT3 — Supplementary Data 

# Asymmetric transcriptomic signatures between the cob and florets in the maize ear under optimal- and low-nitrogen conditions at silking, and functional characterization of amino acid transporters ZmAAP4 and ZmVAAT3

## Supplementary Data

Data files

- Supplementary Data - Supplementary Data
- Supplementary Data - Supplementary Data
- Supplementary Data - Supplementary Data
- Supplementary Data - Supplementary Data
- Supplementary Data - Supplementary Data
