# Supplementary material for: Asymmetric transcriptomic signatures between the cob and florets in the maize ear under optimal- and low-nitrogen conditions at silking, and functional characterization of amino acid transporters ZmAAP4 and ZmVAAT3
Source: J Exp Bot. 2015 Jul 1;66(20):6149–66. doi: 10.1093/jxb/erv315 (PMC4588875; doi:10.1093/jxb/erv315)
Supplement: Supplementary Data [file supp_erv315_jexbot146183_file001.pdf]

Fig.S1 Metabolism overview of the differentially expressed genes in the cob (A) and florets (B) under LN. The red and blue colors indicated up- or down-regulated expression under LN compared to control, respectively.

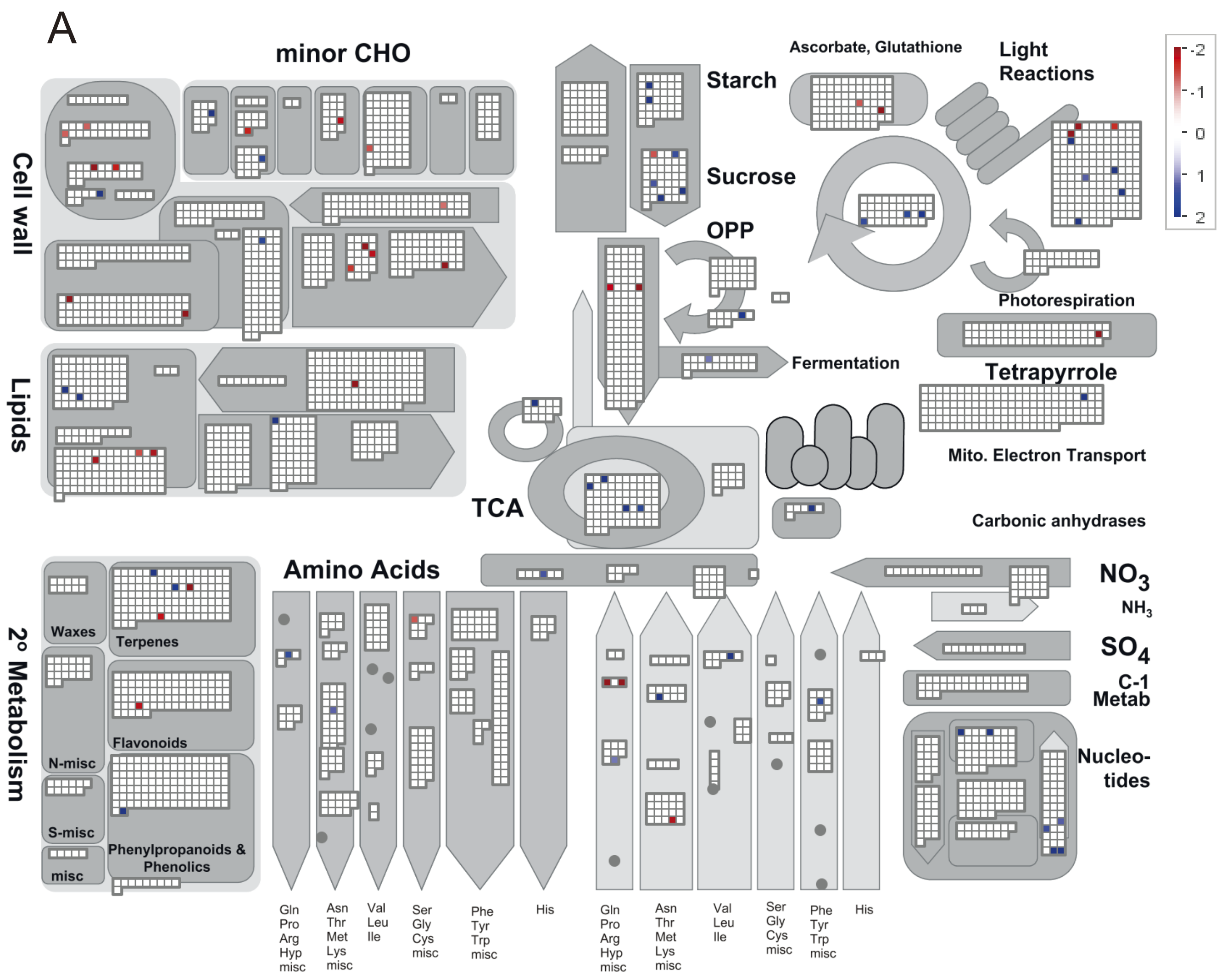

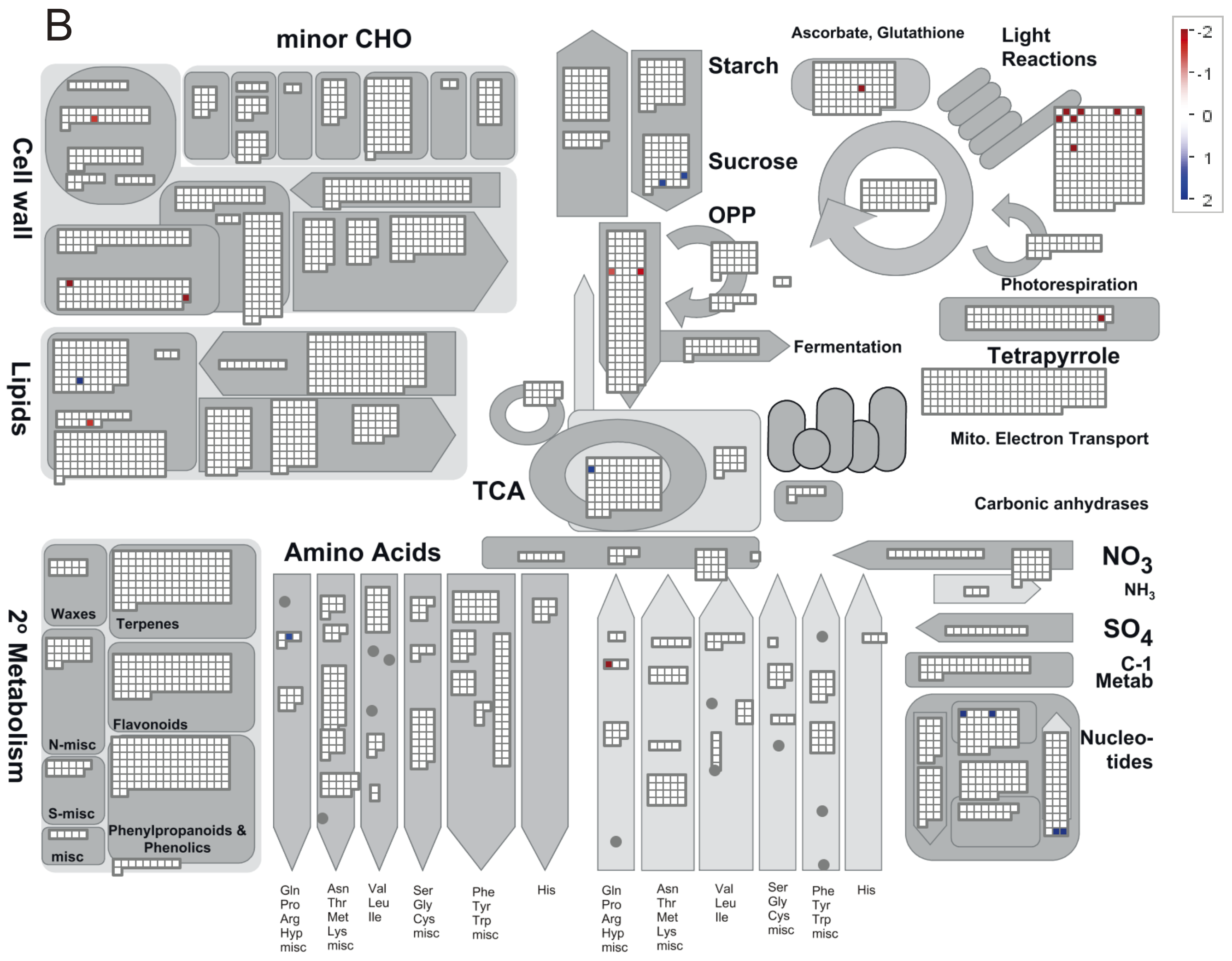

Fig.S2 Transcription levels of *ZmAAP4* and *ZmVAAT3* in gene overexpression lines, with *AtTub4* as the control (Pfaffl, 2001). #1, #2, and #3 denoted three independent transgenic lines.

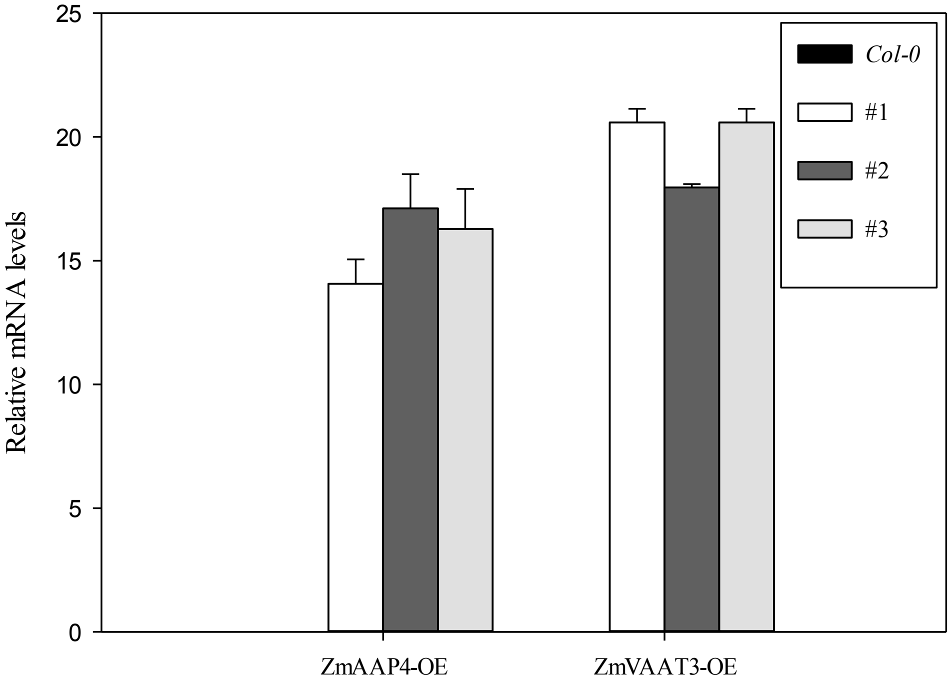

Table S1. *Fertilization practice among different nutrient treatments.*

	**Before sowing**			**V8**	**V12**		**Total (kg ha^{-1})**		
**Treatment**	**N**	**P_2O_5**	**K_2O**	**N**	**N**	**K_2O**	**N**	**P_2O_5**	**K_2O**
ON	60	135	80	120	70	40	250	135	120
LN		135	80			40	0	135	120
LP	60		80	120	70	40	250		120
LK	60	135		120	70		250	135	

Table S2. *Primer sequences used for qRT- PCR.*

Gene ID	Forward primer (5'-3')	Reverse primer (5'-3')
GRMZM2G432926	AACCAGCGAAAGGCGTGGGAGG	AAACTTGGAATGGAAGCCGATGAAA
GRMZM2G072322	GCCACCAGAGTGATGTTTGA	GGGACGAGTAGCCGTTGAGC
GRMZM2G400156	TGCTGTGGATGCTGGAGAAC	AAGAATGCCGAACGAAATGA
GRMZM2G062129	TCCAGGGATACTGAGGATGA	AGGGAGCAGACTTCTATTTACAC
GRMZM2G142680	GTACCATCGTCCATCAATCCC	CGACAACGACATCAACTACCG
GRMZM2G001799	TGGACCAGATCGGCAAGGAATG	TGGCGACACGCACAACTCAAAA
GRMZM2G076345	CGACTACTACGCCTGCTGAACTGC	AAATCAAACCAACGAGCCAAAGC
GRMZM2G437575	TGAAGCCGTTCGTGATTCCAAA	TCGTTCGTCACCACATCCCACA
GRMZM2G318780	GCGGCAGACGGTAGCCAATAA	CAAGGAGATGCTGGGCAACAGAG
GRMZM2G311182	TGCCTGGTCTGTACCGAGTTG	GGAGAAGAGGATGGGCTTTGA
GRMZM2G081192	CTCAGCGACGGCTACTCCAA	ATGAACACGGCGAACCCAAT
GRMZM2G051619	CGTGGAGGGAGTACAAGAA	GATGTGGTGCGACGATAGA
GRMZM2G112377	TCCTTTCTGTCTGGTGCTTGTG	ACGGTGACCTCCGGTTATTTAG
GRMZM2G161905	ACGAGTATAGATGGCAAATGGGTCA	TTGGCAAGGAGACGAACATAAGAAT

Table S3. *Total tags and genes generated by DGE sequencing.*

	Protein coding		Pseudogene		Transposable element	
	**Tags**	**Genes**	**Tags**	**Genes**	**Tags**	**Genes**
Sense	41,751,419	32,805	236,951	1,990	468,513	1,974
Antisense	10,589,158	29,065	828,149	1,976	226,330	1,700
Total	52,340,577	61,870	1,065,100	3,966	694,843	3,674

Table S4. *DGE data verification by qRT- PCR.*

Gene ID	Fold change (Cob vs Floret)		Annotation
	DGE	qRT-PCR	
GRMZM2G134888	5.0	4.5	Amino acid transporter family protein
GRMZM2G162893	15.2	16.3	Ethylene-responsive protein -related
GRMZM2G413887	6.5	6.2	GRAM domain-containing protein / ABA-responsive protein-related
GRMZM2G475683	8.9	8.8	Auxin-responsive family protein
GRMZM2G108537	20	19.2	Nodulin MtN21 family protein
GRMZM2G179349	43.1	46.6	Nodulin MtN3 family protein
GRMZM2G045171	15.0	16.7	SUS6, ATSUS6 | SUS6 (Sucrose Synthase 6)
GRMZM2G085199	8.5	8.7	Ethylene-responsive protein -related
GRMZM2G078024	52.5	54.6	PROT1, ATPROT1 | PROT1 (Proline Transporter 1); L-proline transmembrane transporter
GRMZM2G019974	38.6	41.4	ATOCT2 (*Arabidopsis Thaliana* Organic Cation/Carnitine Transporter 2)
GRMZM2G432926	12.7	13.8	PIP2B, PIP2;2 | PIP2B (Plasma Membrane Intrinsic Protein 2); water channel
GRMZM2G113167	9.3	9.1	GRAM domain-containing protein / ABA-responsive protein-related
GRMZM2G175140	0.01	0.01	ATAMT1;2, AMT1;2 | ATAMT1;2 (Ammonium Transporter 1;2)
GRMZM2G080843	0.01	0.01	Amino acid transporter family protein
GRMZM2G453565	0.1	0.1	AtGH9C3 | AtGH9C3 (Arabidopsis thaliana glycosyl hydrolase 9C3); carbohydrate binding
GRMZM2G088861	0.01	0.03	Leucine-rich repeat protein, putative
GRMZM2G005284	0.01	0.02	ARF10 | ARF10 (Auxin Response Factor 10)
GRMZM2G326707	0.1	0.12	ATPT2, PHT1;4 | ATPT2 (Arabidopsis Thaliana Phosphate Transporter 2); carbohydrate transmembrane transporter
GRMZM2G008792	0.2	0.11	ATCKX6, CKX6, ATCKX7 | CKX6 (Cytokinin oxidase/dehydrogenase 6)
GRMZM2G333980	0.2	0.18	Polygalacturonase Inhibiting Protein 1

Table S9. *Phenotypes of ZmAAP4-OE seedlings 12 days after transfer onto ATS medium supplemented with different amino acids and 3 mM NO_3^-*

Treatment	Primary root length (cm)				Average rosette leaves per plant			
	Col-0	**ZmAAP4-OE-1**	**ZmAAP4-OE-2**	**ZmAAP4-OE-3**	***Col-0***	**ZmAAP4-OE-1**	**ZmAAP4-OE-2**	**ZmAAP4-OE-3**
3 mM NO_3^-	9.78 ± 0.11	9.92 ± 0.12	9.87 ± 0.21	9.83 ± 0.08	10.72 ± 0.11	10.81 ± 0.13	10.91 ± 0.21	10.78 ± 0.14
L-Gly (3 mM)	5.22 ± 0.12	4.12 ± 0.10*	4.32 ± 0.12*	4.25 ± 0.14*	7.94 ± 0.24	7.07 ± 0.13 *	7.12 ± 0.17*	6.94 ± 0.14*
L-Ala (3 mM)	7.50 ± 0.12	8.10 ± 0.13	8.10 ± 0.12	8.28 ± 0.12	9.08 ± 0.14	7.24 ± 0.17**	7.43 ± 0.13**	7.33 ± 0.13**
L-Val (300 µM)	6.71 ± 0.12	2.00 ± 0.13**	2.10 ± 0.11**	2.12 ± 0.12**	9.12 ± 0.14	7.33 ± 0.17**	7.25 ± 0.15**	7.33 ± 0.13**
L-Leu (300 µM)	3.52 ± 0.13	2.73 ± 0.12**	2.63 ± 0.14**	2.71 ± 0.14**	9.33 ± 0.11	9.12 ± 0.12	9.22 ± 0.14	9.13 ± 0.15
L-Ile (3 mM)	3.0 ± 0.12	0.9 ± 0.0**	1.1 ± 0.0**	0.9 ± 0.0**	11.11 ± 0.11	9.07 ± 0.15**	9.33 ± 0.17**	9.42 ± 0.15**
L-Ser (300 µM)	5.13 ± 0.12	5.53± 0.11	5.61± 0.11	5.54± 0.13	9.15± 0.13	7.22± 0.13**	7.32± 0.13**	7.41± 0.13**
L-Cys (3 mM)	6.10 ± 0.12	1.20 ± 0.0**	1.24 ± 0.12**	1.26 ± 0.0**	9.24 ± 0.14	7.12 ± 0.17**	7.16 ± 0.17**	7.33 ± 0.17**
L-Thr (300 µM)	4.82 ± 0.12	2.10 ± 0.12**	2.00 ± 0.10**	2.10 ± 0.10**	8.12 ± 0.11	7.09 ± 0.13*	7.23 ± 0.17*	7.21 ± 0.15*
L-Met (300 µM)	3.50 ± 0.12	1.00 ± 0.0**	1.00 ± 0.0**	1.00 ± 0.0**	10.23 ± 0.14	11.12 ± 0.17*	11.21 ± 0.14*	11.33 ± 0.15*
L-Pro (100 µM)	9.10 ± 0.12	8.32 ± 0.13	8.40 ± 0.12	8.28 ± 0.12	8.13 ± 0.11	7.33 ± 0.15**	7.31 ± 0.17**	7.15 ± 0.13**
L-Phe (300 µM)	6.10 ± 0.12	1.72 ± 0.11**	1.70 ± 0.11**	1.60 ± 0.10**	9.31 ± 0.14	7.24 ± 0.17**	7.33 ± 0.13**	7.13 ± 0.15**
L-Tyr (3 mM)	5.12 ± 0.12	5.22 ± 0.15	5.23± 0.21	5.33 ± 0.15	6.12 ± 0.22	6.17 ± 0.12	6.24 ± 0.24	6.31 ± 0.22
L-Trp (3 mM)	2.32 ± 0.12	2.13 ± 0.12	1.94 ± 0.12	2.12 ± 0.12	6.11 ± 0.23	4.21 ± 0.22**	4.05 ± 0.21**	4.13 ± 0.16**
L-His (300 µM)	4.22 ± 0.12	2.50 ± 0.12**	2.57 ± 0.12**	2.60 ± 0.10**	9.12 ± 0.11	7.16 ± 0.15**	7.32 ± 0.14**	7.08 ± 0.14**
L-Lys (300 µM)	2.30 ± 0.11	1.20 ± 0.08*	1.23 ± 0.12*	1.21 ± 0.21*	8.33 ± 0.14	7.80 ± 0.17**	7.92 ± 0.17**	7.92 ± 0.15**
L-Arg (300 µM)	7.21 ± 0.12	1.42 ± 0.14**	1.52 ± 0.13**	1.42 ± 0.12**	9．33 ± 0.14	7．22 ± 0.14**	7．12 ± 0.14**	7．32 ± 0.14**
L-Asp (3 mM)	7.10 ± 0.12	7.10 ± 0.10	7.12 ± 0.10	7.12 ± 0.13	6.13 ± 0.16	6.15 ± 0.132	6.25 ± 0.21	6.35 ± 0.22
L-Glu (3 mM)	12.10 ± 0.13	10.5 ± 0.10**	10.61 ± 0.10**	10.58 ± 0.13**	8.24 ± 0.11	9.08 ± 0.12*	9.16 ± 0.17*	9.33 ± 0.15*
L-Asn (300 µM)	8.40 ± 0.12	9.00 ± 0.13	9.10 ± 0.10	9.12 ± 0.13	9.24 ± 0.14	10.10 ± 0.17*	10.12 ± 0.17*	10．00 ± 0.17*
L-Gln (3 mM)	12.06 ± 0.12	10.00 ± 0.13**	10.10 ± 0.12**	10.08 ± 0.10**	8.11 ± 0.12	7.12 ± 0.22*	7 .25 ± 0.12*	7.33 ± 0.12*
GABA (3 mM)	7.20 ± 0.12	6.7 0± 0.12	6.60 ± 0.10	6.70 ± 0.10	9.12 ± 0.14	7.12 ± 0.12**	7.25 ± 0.00**	7.08 ± 0.0-0**
L-Cit (100 µM)	9.21 ± 0.21	6.32 ± 0.12**	6.41 ± 0.15**	6.62 ± 0.20**	9.24 ± 0.08	9.15 ± 0.10	9.08 ± 0.12	9.10 ± 0.22

Treatment	Rosette diameter (cm)				Fresh weight per plant (g)			
	Col-0	ZmAAP4-OE-1	ZmAAP4-OE-2	ZmAAP4-OE-3	*Col-0*	ZmAAP4-OE-1	ZmAAP4-OE-2	ZmAAP4-OE-3
3 mM NO_3^-	2.3 ± 0.1	2.2 ± 0.1	2.3 ± 0.1	2.3 ± 0.1	0.0320 ± 0.001	0.0335 ± 0.0010	0.0348 ± 0.0010	0.0332 ± 0.0010
L-Gly (3 mM)	1.5± 0.1	0.9± 0.1*	0.9± 0.1*	0.9± 0.1*	0.0120 ± 0.003	0.0067 ± 0.0003**	0.0064 ± 0.0002**	0.0063 ± 0.0003**
L-Ala (3 mM)	2.1 ± 0.1	2.7 ± 0.1**	2.6 ± 0.1**	2.7 ± 0.1**	0.0280 ± 0.001	0.0345 ± 0.0010**	0.0348 ± 0.0010**	0.0342 ± 0.0010**
L-Val (300 µM)	1.6 ± 0.1	0.8 ± 0.0**	0.8 ± 0.0**	0.8 ± 0.0**	0.0253 ± 0.001	0.0190 ± 0.001**	0.0194 ± 0.001**	0.0189 ± 0.001**
L-Leu (300 µM)	2.2± 0.1	2.2± 0.1	2.2± 0.1	2.2± 0.1	0.0280 ± 0.001	0.0367± 0.002**	0.0353± 0.002**	0.0345± 0.001**
L-Ile (3 mM)	2.3 ± 0.1	0.8 ± 0.0**	0.8 ± 0.0**	0.8 ± 0.0**	0.0250 ± 0.001	0.0073 ±0.0003***	0.0070 ± 0.0004***	0.0075 ±0.0003***
L-Ser (300 µM)	2.2 ± 0.1	1.8 ± 0.1**	1.7 ± 0.1**	1.6 ± 0.1**	0.0300 ± 0.001	0.0257 ± 0.001**	0.0244± 0.001**	0.0252± 0.001**
L-Cys (3 mM)	2.0 ± 0.1	1.0 ± 0.0**	1.0 ± 0.0**	0.9 ± 0.0**	0.0340 ± 0.001	0.0107 ± 0.002**	0.0112 ± 0.001**	0.0102 ± 0.002**
L-Thr (300 µM)	1.0 ± 0.1	0.9± 0.0	0.9± 0.0	0.9± 0.0	0.0233 ± 0.001	0.0100 ± 0.001 **	0.0100 ± 0.001 **	0.0100 ± 0.001 **
L-Met (300 µM)	2.7 ± 0.1	1.6 ± 0.1**	1.5 ± 0.1**	1.6 ± 0.1**	0.0267 ± 0.001	0.0143 ± 0.001 **	0.0142 ± 0.001 **	0.0146 ± 0.001**
L-Pro (100 µM)	1.6 ± 0.1	1.6 ± 0.1	1.6 ± 0.1	1.5 ± 0.1	0.029 ± 0.001	0.020 ± 0.002 **	0.021 ± 0.001**	0.022 ± 0.001 **
L-Phe (300 µM)	1.7 ± 0.1	1.2 ± 0.1*	1.1 ± 0.1*	1.2 ± 0.1*	0.0213 ± 0.001	0.0100 ± 0.001**	0.0120 ± 0.001**	0.0110 ± 0.001**
L-Tyr (3 mM)	1.6 ± 0.1	1.2± 0.1*	1.2± 0.1*	1.2± 0.1*	0.0067± 0.0002	0.0020 ± 0.0001**	0.0020 ± 0.0001**	0.0020 ± 0.0001**
L-Trp (3 mM)	0.8± 0.0	0.6 ± 0.0*	0.6 ± 0.0*	0.6 ± 0.0*	0.0067± 0.0002	0.0020 ± 0.0001**	0.0020 ± 0.0001**	0.0020 ± 0.0001**
L-His (300 µM)	1.7 ± 0.1	1.5 ± 0.1	1.5 ± 0.1	1.5 ± 0.1	0.0177 ± 0.001	0.0043 ±0.0003**	0.0045 ±0.0003**	0.0042 ±0.0003**
L-Lys (300 µM)	1.4 ± 0.0	0.5 ± 0.0**	0.5 ± 0.0**	0.5 ± 0.0**	0.0237± 0.0004	0.0057 ±0.0003**	0.0059 ±0.0004**	0.0060 ±0.0004**
L-Arg (300 µM)	2.2 ± 0.1	0.8 ± 0.0**	0.8 ± 0.0**	0.8 ± 0.0**	0.0333 ± 0.0004	0.0107 ± 0.0003**	0.0114 ± 0.0004**	0.0118 ± 0.0004**
L-Asp (3 mM)	2.0 ± 0.1	2.0 ± 0.1	2.0 ± 0.1	1.9 ± 0.1	0.021 ± 0.001	0.022 ± 0.001	0.023 ± 0.001	0.023 ± 0.001
L-Glu (3 mM)	3.1 ± 0.1	2.5 ± 0.1**	2.4 ± 0.1**	2.5 ± 0.1**	0.03533 ± 0.001	0.0233 ± 0.001**	0.0244 ± 0.001**	0.0221 ± 0.001**
L-Asn (300 µM)	1.6 ± 0.1	1.6 ± 0.0	1.6 ± 0.0	1.6 ± 0.0	0.0183 ± 0.001	0.0187 ± 0.001	0.0185 ± 0.001	0.0183 ± 0.001
L-Gln (3 mM)	1.5 ± 0.1	1.5 ± 0.1	1.5 ± 0.1	1.5 ± 0.0	0.0086 ± 0.0002	0.0090 ± 0.0002	0.0092 ± 0.0001	0.0090 ± 0.0002
GABA (3 mM)	1.5± 0.0	1.1 ± 0.0**	1.1 ± 0.0**	1.0 ± 0.0**	0.0123 ± 0.001	0.0080 ±0.0002*	0.0080±0.0003*	0.0080 ±0.0003*
L-Cit (100 µM)	1.6 ± 0.1	1.4 ± 0.1	1.5 ± 0.1	1.4 ± 0.1	0.028 ± 0.001**	0.023 ± 0.001**	0.023 ±0.001**	0.024 ± 0.002**

Values are means ± SD; n = 12 individuals. Asterisks indicate significant differences as follows: * $P < 0.05$, ** $P < 0.01$

Table S10. *Phenotypes of ZmVAAT3-OE seedlings 12 days after transfer onto ATS medium supplemented with different amino acids and 3 mM NO_3^-*

Treatment	Primary root length (cm)				Average rosette leaves per plant			
	Col-0	ZmVAAT3-OE-1	ZmVAAT3-OE-2	ZmVAAT3-OE-3	*Col-0*	ZmVAAT3-OE-1	ZmVAAT3-OE-2	ZmVAAT3-OE-3
3 mM NO_3^-	10.12 ± 0.12	10.21 ± 0.13	10.22 ± 0.12	10.13 ± 0.12	8.42 ± 0.14	8.49 ± 0.22	8.33 ± 0.12	8.52 ± 0.08
L-Gly (3 mM)	5.11 ± 0.21	5.0 ± 0.22	5.12 ± 0.21	5.11 ± 0.22	8.22 ± 0.24	7.11 ± 0.13 *	7.34 ± 0.17*	7.23 ± 0.14*
L-Ala (3 mM)	8.13 ± 0.11	13.22 ± 0.21**	13.11 ± 0.2**	13.0 1± 0.31**	8 .33 ± 0.14	7.36 ± 0.12*	7 .24 ± 0.16*	7.33 ± 0.21*
L-Val (300 µM)	3.51 ± 0.22	5.12 ± 0.21*	5.21 ± 0.12*	5.2 2± 0.22*	8.11 ± 0.12	7 .22 ± 0.22*	7.33 ± 0.14*	7.14 ± 0.15*
L-Leu (300 µM)	3.32 ± 0.21	10.52 ± 0.23**	10.53 ± 0.23**	10. 43 ± 0.24**	8.23 ± 0.12	8.31 ± 0.13	8.25 ± 0.17	8.35 ± 0.13
L-Ile (3 mM)	3.71 ± 0.21	3.61 ± 0.12	3.61 ± 0.31	3.51 ± 0.22	7.52 ± 0.22	5.34 ± 0.23**	5.32 ± 0.24**	5.31 ± 0.14**
L-Ser (300 µM)	5.81 ± 0.21	3.52 ± 0.12**	3.42 ± 0.16**	3.51 ± 0.21**	8.41 ± 0.14	6.32 ± 0.22**	6.53 ± 0.24**	6.42± 0.14**
L-Cys (3 mM)	6.22 ± 0.22	5.51 ± 0.23*	5.42 ± 0.24*	5.52 ± 0.16*	8.21 ± 0.11	7.24 ± 0.12*	7.33 ± 0.15*	7.50 ± 0.19*
L-Thr (300 µM)	4.22 ± 0.12	3.53 ± 0.13*	3.52 ± 0.12*	3.43 ± 0.12*	6.32 ± 0.22	5.25 ± 0.14**	5.33 ± 0.12**	5.25± 0.17**
L-Met (300 µM)	3.81 ± 0.21	9.53 ± 0.22**	9.41 ± 0.22**	9.52 ± 0.13**	9.21 ± 0.21	7.33 ± 0.14*	7.41 ± 0.13*	7.34 ± 0.12*
L-Pro (100 µM)	10.62 ± 0.22	10.71 ± 0.21	10.61 ± 0.23	10.72 ± 0.22	10.52 ± 0.22	8.23 ± 0.12	8.32 ± 0.23	8.18 ± 0.13
L-Phe (300 µM)	8.21 ± 0.22	11.12 ± 0.22**	11.32 ± 0.21**	11.41 ± 0.22**	8.62 ± 0.21	9.73 ± 0.23*	9.81 ± 0.17*	9.64 ± 0.12*
L-Tyr (3 mM)	5.32 ± 0.14	5.32 ± 0.13	5.43± 0.23	5.43 ± 0.23	6.24 ± 0.22	6.32 ± 0.12	6.34 ± 0.24	6.61 ± 0.22
L-Trp (3 mM)	2.42 ± 0.12	2.03 ± 0.13	1.92 ± 0.13	2.02 ± 0.13	6.51 ± 0.23	4.43 ± 0.22**	4.33 ± 0.21**	4.25 ± 0.16**
L-His (300 µM)	3.00 ± 0.10	2.51 ± 0.22*	2.62 ± 0.12*	2.42 ± 0.13*	8.53 ± 0.21	6.32 ± 0.22**	6.38 ± 0.13**	6.51 ± 0.17**
L-Lys (300 µM)	2.10 ± 0.13	2.03 ± 0.12	2.03 ± 0.12	1.90 ± 0.12	7.23 ± 0.12	5.32 ± 0.23**	5.37 ± 0.22**	5.38 ± 0.12**
L-Arg (300 µM)	8.31 ± 0.21	8.82 ± 0.12	8.9 2± 0.12	8.82 ± 0.12	8.33 ± 0.12	8.24 ± 0.21	8.12 ± 0.11	8.37 ± 0.13
L-Asp (3 mM)	7.72 ± 0.12	7.62 ± 0.23	7.73 ± 0.23	7.71 ± 0.13	6.33 ± 0.16	6.25 ± 0.12	6.32 ± 0.21	6.33 ± 0.22
L-Glu (3 mM)	8.52 ± 0.18	8.62 ± 0.25	8.72 ± 0.25	8.64 ± 0.25	9.25 ± 0.22	7.09 ± 0.12**	7.33 ± 0.12**	7.42 ± 0.15**
L-Asn (300 µM)	8.62 ± 0.22	11.56 ± 0.23**	11.65 ± 0.34**	11.44 ± 0.24**	8.23 ± 0.16	8.12± 0.19	8.09± 0.21	8.0± 0.13
L-Gln (3 mM)	13.12 ± 0.21	11.61 ± 0.22*	11.52 ± 0.21*	11.51 ± 0.23*	7.22 ± 0.12	8.32 ± 0.22*	8 .12 ± 0.12*	8.22 ± 0.12*
GABA (3 mM)	9.12 ± 0.12	7.24 ± 0.11**	7.13 ± 0.11**	7.32 ± 0.12**	8 .25 ± 0.15	8.25 ± 0.21	8.33 ± 0.13	8.66 ± 0.12
L-Cit (100 µM)	9.22 ± 0.22	6.61 ± 0.21**	6.57 ± 0.22**	6.73 ± 0.22**	8.24 ± 0.13	6.23 ± 0.12**	6.27 ± 0.13**	6.21 ± 0.17**

Treatment	Rosette diameter (cm)				Fresh weight per plant (g)			
	Col-0	ZmVAAT3-OE -1	ZmVAAT3-OE -2	ZmVAAT3-OE -3	*Col-0*	ZmVAAT3-OE -1	ZmVAAT3-OE -2	ZmVAAT3-OE -3
3 mM NO_3^-	1.7 ±0.00	1.7 ±0.00	1.7 ±0.00	1.7 ±0.00	0.0213 ± 0.001	0.0227 ± 0.001	0.0210 ± 0.001	0.0228 ± 0.002
L-Gly (3 mM)	1.4 ± 0.1	1.1 ±0.0**	1.1 ±0.0**	1.1 ±0.0**	0.0100 ± 0.003	0.0097 ± 0.0003	0.0094 ± 0.0002	0.0093 ± 0.0003
L-Ala (3 mM)	1.2 ± 0.10	1.6 ± 0.10**	1.6 ± 0.10**	1.7 ± 0.10**	0.0210 ± 0.002	0.0223 ± 0.003	0.0225 ± 0.003	0.0224± 0.004
L-Val (300 μM)	1.8 ± 0.1	1.5 ± 0.0**	1.5 ± 0.0**	1.5 ± 0.0**	0.0153 ± 0.002	0.0155 ± 0.002	0.0152 ±0.003	0.0155 ± 0.003
L-Leu (300 μM)	1.6 ± 0.1	1.5 ± 0.10	1.5 ± 0.10	1.5 ± 0.10	0.0253 ± 0.001	0.0257 ± 0.001	0.0240 ± 0.001	0.0248 ± 0.002
L-Ile (3 mM)	1.6 ± 0.20	0.5 ± 0.00**	0.5 ± 0.00**	0.5 ± 0.00**	0.0220 ± 0.003	0.004 ± 0.001**	0.004 ± 0.001**	0.004 ± 0.001**
L-Ser (300 μM)	1.5 ± 0.1	1.1 ± 0.1**	1.1 ± 0.0**	1.1 ± 0.1**	0.0127 ± 0.0004	0.0057 ± 0.0003**	0.0056 ±0.0002**	0.0053 ± 0.0003**
L-Cys (3 mM)	1.8 ± 0.10	1.9 ± 0.10	1.9 ± 0.10	2.0 ± 0.10	0.0303 ± 0.0003	0.0190 ± 0.0002**	0.0188 ± 0.0002**	0.0190 ± 0.0002**
L-Thr (300 μM)	1.5 ± 0.10	1.4 ± 0.10	1.4 ± 0.10	1.4 ± 0.10	0.0173 ± 0.0001	0.0157 ± 0.0001	0.0156 ± 0.0002	0.0157 ± 0.0001
L-Met (300 μM)	1.5 ± 0.1	1.3 ± 0.1	1.3 ± 0.10	1.3 ± 0.10	0.0103 ± 0.0003	0.0173 ± 0.0002**	0.0162 ± 0.0003**	0.0170 ± 0.0003**
L-Pro (100 μM)	1.8 ± 0.2	1.9 ± 0.1	1.9 ± 0.1	1.8 ± 0.1	0.018 ± 0.001	0.027 ± 0.001**	0.025 ± 0.002**	0.027 ± 0.001**
L-Phe (300 μM)	1.3 ± 0.10	1.3 ± 0.0	1.3 ± 0.10	1.2 ± 0.10	0.0123 ± 0.001	0.0163 ±0.001*	0.0162 ±0.001*	0.0161 ±0.001*
L-Try (3 mM)	2.1 ± 0.10	1.8 ± 0.10	1.8 ± 0.20	1.8 ± 0.10	0.0117 ±0.0003	0.0114 ±0.0003	0.0115 ±0.0003	0.0111 ±0.0003
L-Trp (3 mM)	0.7 ± 0.00	0.3 ± 0.00**	0.3 ± 0.00**	0.3 ± 0.00**	0.0067± 0.0002	0.0020 ± 0.0001**	0.0020 ± 0.0001**	0.0020 ± 0.0001**
L-His (300 μM)	1.1 ± 0.1	0.8 ± 0.0*	0.8 ± 0.0*	0.8 ± 0.0*	0.0111 ± 0.001	0.0053 ± 0.0002**	0.0050 ± 0.0002**	0.0058 ± 0.0001**
L-Lys (300 μM)	1.0 ± 0.10	0.7 ± 0.00**	0.7 ± 0.00**	0.7 ± 0.00**	0.029 ± 0.001**	0.006 ± 0.0**	0.006 ± 0.0**	0.006 ± 0.0**
L-Arg (300 μM)	2.0 ± 0.10	2.0 ± 0.10	2.0 ± 0.10	1.8 ± 0.10	0.0200 ± 0.0002	0.0183 ± 0.0002	0.0181 ± 0.0002	0.0184 ± 0.0002
L-Asp (3 mM)	1.8 ± 0.10	1.6 ± 0.20	1.6 ± 0 .10	1.6 ± 0.10	0.024 ± 0.001	0.022 ± 0.001	0.023 ± 0.001	0.023 ± 0.001
L-Glu (3 mM)	2.3 ± 0.20	1.8 ± 0.1**	1.8 ± 0.10**	1.8 ± 0.10**	0.0277 ± 0.0002	0.0190 ± 0.0002**	0.0182 ± 0.0002**	0.0193 ± 0.0002**
L-Asn (300 μM)	1.4 ± 0.10	1.5 ± 0.10	1.5 ± 0.10	1.5 ± 0.10	0.0140 ± 0.0002	0.0173 ± 0.0003*	0.0171 ± 0.0002*	0.0178 ± 0.0002*
L-Gln (3 mM)	2.0 ± 0.20	1.5 ± 0.10**	1.5 ± 0.10**	1.5 ± 0.10**	0.0136 ± 0.0002	0.0100 ± 0.0002*	0.0102 ± 0.0001*	0.0100 ± 0.0002*
GABA (3 mM)	1.9 ± 0.10	1.9 ± 0.20	1.8 ± 0.10	1.8 ± 0.10	0.0170 ± 0.0003	0.0130 ± 0.0003*	0.0130 ± 0.003*	0.01300 ± 0.0003*
L-Cit (100 μM)	1.4 ± 0.1	0.9 ± 0.10**	0.9 ± 0.00**	0.9 ± 0.10**	0.0113 ± 0.0004	0.0120 ± 0.0002	0.0094 ± 0.0002	0.0092 ± 0.0002

Values are means ± SD; n = 12 individuals. Asterisks indicate significant differences as follows: * $P < 0.05$, ** $P < 0.01$

Table S11. *Effects of different amino acids on growth parameters of ZmAAP4-OE and ZmVAAT3-OE arabidopsis seedlings*

**Parameter**	**Effect**	**Amino acids**	
		**ZmAAP4-OE**	**ZmVAAT3-OE**
Root length	Positive	-	Leu, Ala, Val, Phe, Met, Arg, Asn, Glu
	Negative	Gly, Val, Cys, Thr, Cit, Leu, Ile, Met, Pro, Trp, Phe, His, Lys, Arg, Glu, Gln, GABA	Ser, Cys, Thr, Trp, Lys, Gln, Cit, GABA, His
Rosette leaf number	Positive	Met, Tyr, Asp, Glu Asn	Leu, Phe, Tyr, Gln,
	Negative	Gly, Ala, Val, Leu, Ile, Ser, Cys, Thr, Pro, Phe, Trp, His, Lys, Arg, Gln, Cit, GABA	Gly, Ala, Val, Ile, Ser, Cys, Thr, Met, Pro, Trp, His, Lys, Arg, Glu, Asn, Cit
Rosette diameter	Positive	Ala	Ala
	Negative	Gly, Val, Ile, Ser, Cys, Met, Phe, Tyr, Trp, Lys, Arg, Glu, GABA	Gly, Val, Ile, Ser, Tyr, Trp, His, Lys, Glu, Gln, Cit
Fresh weight	Positive	Ala, Leu	Met, Pro, Phe, Asn
	Negative	Gly, Val, Ile, Ser, Cys, Thr, Met, Pro, Phe, Tyr, Trp, His, Lys, Arg, Glu, GABA, Cit	Ile, Ser, Cys, Trp, His, Lys, Arg, Glu, Gln, GABA

Supplementary dataset 1. Coding sequences and functional domains of ZmAAP4 and ZmVAAT3

Coding sequences of two amino acid transporters (the sequence from the start codon "ATG" to the stop codon "TGA" or "TAG" were subcloned into the overexpression vector for gene overexpression) and illustration of their functional domains (Tusnády and Simon, 1998; 2001).

ZmAAP4 **(GRMZM2G110195_T01)**:
ATGGCGGAGAACAACGTCGTGGCCACGTACTACTACCCGACGGCAGCGCC
GGCGGCCATGGAGGTCTGCGGCGCGGAGCTCGGCCAGGGCAAGCCCGAC
AAGTGCTTCGACGACGATGGCCGCCCCAAGCGCAATGGGACGATGTGGAC
GGCGAGCGCGCACATCATCACGGCGGTGATCGGCTCCGGGGTGCTCTCGC
TGGGGTGGGCCATCGCGCAGCTCGGCTGGGTGGCCGGACCCGTCGTCATG
CTGCTCTTCTCGCTCGTCACCTACTACACCTCGTCGCTGCTCGCAGACTGC
TACCGCTCCGGCGACCCCAGCACCGGCAAGCGGAACTACACCTACATGGA
CGCCGTCAACGCGAACCTCAGTGGCATCAAGGTCCAGATCTGCGGGTTCC
TGCAGTACGCCAACATCGTGGGCGTGGCCATCGGCTACACCATCGCTGCC
TCCATTAGCATGCTCGCGATCAGGAGGGCCAACTGCTTCCACCAGAAGGG
ACACGGCAACCCCTGCAAGATCTCCAGCACGCCCTACATGATCATCTTCG
GCGTGGCGGAGATCTTCTTCTCGCAGATCCCGGACTTCGACCAGATCTCCT
GGCTCTCCATCCTCGCCGCCGTCATGTCCTTCACCTACTCCTCCATTGGGC
TCGGCCTGGGCGTCGTCCAAGTCATCGCGAACAGAGGCGTGCAGGGCAGC
CTGACCGGCATCACCATCGGCGTGGTGACCCCGATGGACAAGGTGTGGCG
CAGCCTCCAGGCGTTCGGCGACGTCGCCTTCGCCTACTCCTACTCCCTCAT
CCTGATCGAGATCCAGGACACCATCCGGGCGCCGCCGCCGTCGGAGTCGA
CGGTGATGAAGCGCGCCACGGTGGTGAGCGTGGCGGTCACCACGCTCTTC
TACATGCTGTGCGGCTGCATGGGGTACGCGGCGTTCGGCGACGGCGCGCC
CGGGAACCTCCTCACGGGCTTCGGCTTCTACGAGCCCTTCTGGCTCCTGGA
CGTGGCCAACGCCGCCATCGTGGTCCACCTGGTCGGCGCCTACCAGGTCT
ACTGCCAGCCGCTGTTCGCCTTCGTGGAGAAGTGGGCCGCGCAGCGGTGG
CCGGACTCGGCGTACATCACCGGGGAGGTCGAGGTCCCGCTCCCGCTCCC
GGCGAGCCGGCGGCGGTGCTGCAAGGTGAACCTGTTCCGGGCGACGTGGC
GGACGGCGTTCGTCGTGGCCACGACGGTCGTGTCCATGCTGCTGCCCTTCT
TCAACGACGTGGTGGGCTTCCTGGGCGCGCTCGGCTTCTGGCCGCTCACC
GTCTACTTCCCCGTCGAGATGTACGTGGTGCAGAAGAAGGTGCCGCGGTG
GAGCTCCCGGTGGGTGTGCCTGCAGATGCTCAGCCTCGGCTGCCTCGTCAT
CTCCATCGCCGCCGCAGCCGGGTCCATCGCCGGCATCGCGTCCGACCTCA
AAGTCTACCGCCCGTTCAAGTCCTACTGA

Illustration of 12 transmembrane domains of ZmAAP4. Locations of trans-membrane helices (from left to right): 51-67, 74-90, 139-155, 174-190, 199-223, 230-246, 255-271, 290-309, 336-360, 403-419, 424-440, 459-483.

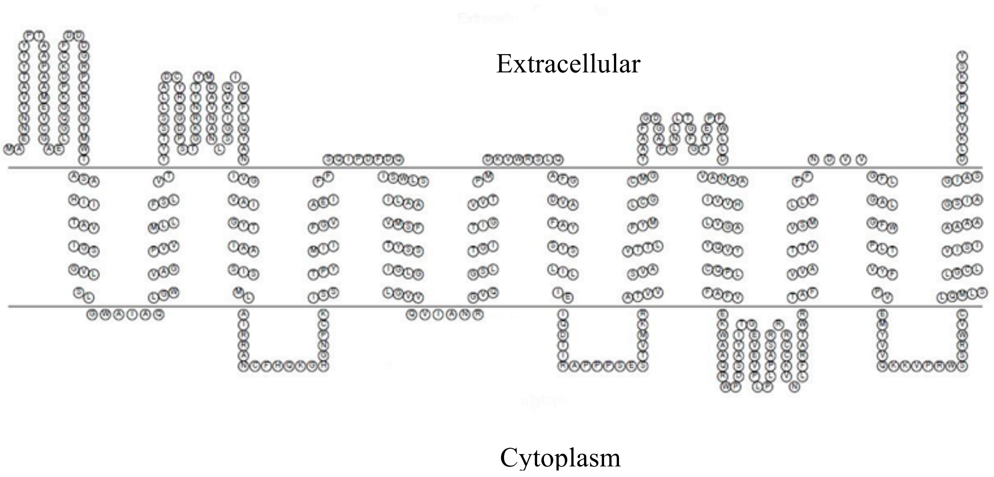

***ZmVAAT3* (GRMZM2G080843_T01)**:
ATGGCGGCGGCGGAGGAGGAGGAGGAGTGGGAGGATGGGCGTTCGGTGA
TGCCGGAGGAGGAGGACGGAGACATCGAGGACGTCCCACCGCCGCCGCT
GCTAGGCAGGAACTTCGGCAGCGAGGACGAGGACGAGGACGAGGCGGGC
TCGTTGAACTCCCAGTCCCAGTGGTGGCCGCGGAGCTTCCGGGAAGCGAC
TGACACCTATACCATTGCCGCGCCCCCGGGCCTCCGTCACCTCGTCGTCGG
TGACTGCAATGGCCTGGGCTCTGACCTGAAGCTGCCTCTTCTGTCGGACAA
GCTAGATGGGAGGCAAGATTCATCCAACAACTTGCTGCCAGTAGCAGACC
CCTTCGGTTCAGTGCTAAGCGACGGGAGATTCTCAGATAGAGTGCAACAA
GCCCCAACCACTCCAGGATGCAATCTGACGCAAACCGTGTTCAATGGGGT
CAACGTGCTTGCTGGCATTGGCATTTTCTCTGCTCCATACACGATTCGTGA
GGCCGGATGGGCCAGCCTTGTGGTTCTGGCTTTTTTCGCTGTGGTGTGCTG
CTACACTGGAGTTCTTCTAAAGTACTGCTTTGAGAGCAAAGATGGTGTCA
AAACTTTCCCAGACATTGGGGAGCTTGCCTTTGGAAGGATCGGCCGGTTT
CTCATTTCTATAGTTCTGTACACCGAGCTCTATTCCTTTTGTGTGGAGTTCG
TTATTCTGGAAGGTGATAACCTGGCATCAATTTTCACGAGCACCACTTTCG
ATTGGAATGGGATTCATGCCGATGGCAGACATTTCTTTGGGATTCTATTTG
CTCTTGTTGTCTTACCCAGCGTGTGGTTGCGAGATCTTCGAGTAATATCTT
ACCTTTCAGTGGGTGGTGTGTTTGCAACTCTTCTGGTTTTCCTATCTGTTGG
TGTAGTTGGTGCTACTGGCAACGTAGGGTTCCACTTGGCAGGGAAAGCAG
TGAAGTGGGATGGTATACCATTTGCAATTGGCATTTATGGTTTTTGCTACG
CTGGACACTCAGTGTTTCCAAATATCTATCAGTCAATGTCCGATCGCACCA
AATTCAACAAGGCACTCTACATATGTTTTGCAATATGCACGACAATTTATG
GCGCTATAGCTGTTATTGGCTACCTCATGTTTGGAGATAAGACTCTCTCAC
AAATAACTTTGAATCTTCCGAAAGATTCATTTGTGGCAAAAGTTGCACTGG
GGACCACGGTGATCATTCCTTTCACAAAATATTCTTTGGTGATAAACCCAT
TGGCTCGGAGTATAGAAGAGTTGCGTCCAGAAGGGTTTCTAACCGATAGA
CTCTTCGCTGTTATGCTGCGTACTGCCATTGTTGCATCAACTCTATGTGTTG
CCTTTCTCCTGCCATTCTTTGGTCTTGTGATGGCTCTCATTGGGTCTCTTCT
CAGTATACTTGTGGCGCTCATAATGCCTGCCCTGTGCTTCCTGAAGATCGC
TCGGAACAAAGCGACGCGCTTGCAGGTCATCGCAAGCGTCGCGACCGTGG
TGCTGGGCTCCGTCTGCGCAGTTCTTGGGACGTACAACTCTATCGCCAAGA
TAGCCGAAAGCTACTAG

Illustration of 11 transmembrane domains of ZmVAAT3. Locations of transmembrane helices (from left to right): 146-165, 170-190, 217-237, 262-278, 287-306, 327-346, 359-381, 402-422, 439-459, 464-487, 500-522.

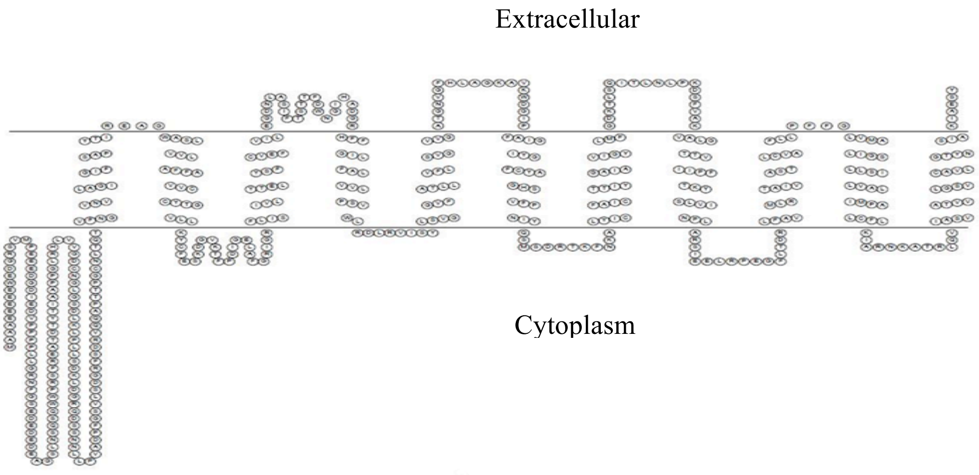

References:

Tusnady GE, Simon I. 1998. Principles governing amino acid composition of integral membrane proteins: application to topology prediction. *Journal of Molecular Biology* **283**, 489-506.

Tusnady GE, Simon I. 2001. The HMMTOP transmembrane topology prediction server. *Bioinformatics* **17**, 849-850.
